# Generic Reporter Sets for Colorimetric Multiplex dPCR Demonstrated with 6-Plex SNP Quantification Panels

**DOI:** 10.3390/ijms25168968

**Published:** 2024-08-17

**Authors:** Maximilian Neugebauer, Silvia Calabrese, Sarah Müller, Truong-Tu Truong, Peter Juelg, Nadine Borst, Tobias Hutzenlaub, Eva Dazert, Nikolas Christian Cornelius von Bubnoff, Felix von Stetten, Michael Lehnert

**Affiliations:** 1 Hahn-Schickard, Georges-Koehler-Allee 103, 79110 Freiburg, Germany; maximilian.neugebauer@hahn-schickard.de (M.N.); silvia.calabrese@hahn-schickard.de (S.C.); muelles5@hs-albsig.de (S.M.); tu.truong@hahn-schickard.de (T.-T.T.); michael.lehnert@hahn-schickard.de (M.L.); 2 Laboratory for MEMS Applications, IMTEK—Department of Microsystems Engineering, University of Freiburg, Georges-Koehler-Allee 103, 79110 Freiburg, Germany; 3 Department of Hematology and Oncology, University Hospital of Schleswig-Holstein, Campus Lübeck, Ratzeburger Allee 160, 23538 Lübeck, Germany; eva.dazert-klebsattel@uksh.de (E.D.); nikolaschristiancornelius.vonbubnoff@uksh.de (N.C.C.v.B.)

**Keywords:** digital PCR (dPCR), mediator probe PCR (MP PCR), generic reporter set, assay development, color compensation, mediator extension assay (MEA), fluorophore combination, oncogenic mutations, *KRAS*, *BRAF*, *NRAS*

## Abstract

Digital PCR (dPCR) is a powerful method for highly sensitive and precise quantification of nucleic acids. However, designing and optimizing new multiplex dPCR assays using target sequence specific probes remains cumbersome, since fluorescent signals must be optimized for every new target panel. As a solution, we established a generic fluorogenic 6-plex reporter set, based on mediator probe technology, that decouples target detection from signal generation. This generic reporter set is compatible with different target panels and thus provides already optimized fluorescence signals from the start of new assay development. Generic reporters showed high population separability in a colorimetric 6-plex mediator probe dPCR, due to their tailored fluorophore and quencher selection. These reporters were further tested using different *KRAS*, *NRAS* and *BRAF* single-nucleotide polymorphisms (SNP), which are frequent point mutation targets in liquid biopsy. We specifically quantified SNP targets in our multiplex approach down to 0.4 copies per microliter (cp/µL) reaction mix, equaling 10 copies per reaction, on a wild-type background of 400 cp/µL for each, equaling 0.1% variant allele frequencies. We also demonstrated the design of an alternative generic reporter set from scratch in order to give detailed step-by-step guidance on how to systematically establish and optimize novel generic reporter sets. Those generic reporter sets can be customized for various digital PCR platforms or target panels with different degrees of multiplexing.

## 1. Introduction

Digital PCR (dPCR) has seen a remarkable surge in its applications in recent years, spanning liquid biopsy [1], single-cell analysis [2] and gene expression studies [3], among many others [4,5]. This progress has been accompanied by the development of commercially available dPCR platforms with an increasing number of detection channels, number of compartments per sample and overall sample throughput [6,7]. This development is largely attributable to the advantages of dPCR over other techniques, including its capacity for absolute quantification of nucleic acid targets, its exceptional sensitivity, its advanced multiplexing capabilities compared to real-time PCR (qPCR) and a certain level of tolerance towards inhibitors [8,9]. However, while dPCR holds great promise, it is important to be aware of the complexities associated with assay development and optimization, as it remains a time-consuming and error-prone process. The performance of fluorescence-probe-based dPCR assays is, for example, reflected in fluorescence signal intensity, detection limits and rain. These parameters are highly dependent on uncontrollable or only partially controllable factors such as target sequences (including primer and probe binding sites), oligonucleotide interactions and fluorescence intensity variations due to non-uniform probe cleavage [10,11,12]. It should be noted that the optimization steps involved in dPCR are significantly different from those of qPCR [13,14,15,16], and therefore, established qPCR assay development guidelines [17] are only partially applicable to dPCR contexts at best. In general, dPCR assays with high degrees of multiplexing are especially complicated to develop, since a higher number of different oligonucleotide sequences could potentially interfere with each other and mutual impairment of parallel reactions occurs regularly. This problem is exacerbated by the development of more recent techniques with higher-order multiplexing capabilities, such as amplitude-based and ratio-based approaches [5]. To unlock the full potential of dPCR, these issues need to be addressed effectively.

**Figure 1:**
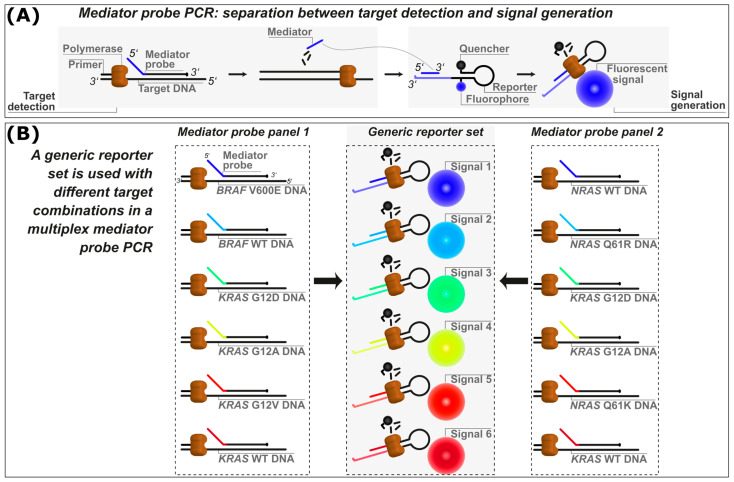
(**A**) Schematic overview of the mediator probe PCR (MP PCR) principle, showing the separation between the target detection and fluorescence signal generation steps. (**B**) Schematic overview of the generic reporter set principle. A generic reporter set with highly distinguishable fluorophore signals is designed and optimized. Each of these reporters needs to be tagged with a specific mediator binding site sequence to allow binding of a specific mediator. Mediator probes (MPs) with target binding sites and mediator sequences that are reverse complementary to the mediator binding sites of the reporter as well as appropriate primer pairs are designed (mediator probe panel 1). The mediators are released after being cleaved from their MP by polymerase and bind the corresponding mediator binding site for signal generation. Several mediator probe panels (e.g., mediator probe panel 2) with the same mediator sequences can be designed for combination with the generic reporter set. WT: wild-type.

In previous publications, our research group has presented mediator probe PCR (MP PCR) [18,19,20,21,22] to tackle the multifaceted challenges posed by assay development within the spectrum of qPCR and dPCR applications. MP PCR succeeded in its aim of separating DNA detection and signal generation steps during PCR to allow optimization of one of these steps without interfering with the other during assay design. The separation of DNA detection and signal generation is achieved through the use of mediator probes, which are not fluorescently labeled but have an oligonucleotide sequence (the mediator) at their 5′-end, which is cleaved from the rest of the probe during target detection and amplification by the polymerase. As a result, the released mediator can bind to a reporter oligonucleotide, labeled with a fluorophore and a quencher, also known as a universal reporter [23], where it will then be extended by the polymerase. As a consequence, the fluorophore and quencher are removed from their initial proximate locations and separated, which leads to a high fluorescence signal increase (Figure 1A). The signal generation of universal reporters can directly be analyzed by mediator extension assays (MEAs) [19] without PCR amplification of the DNA target sequence. Here, in the absence of primers, target and probe, only the mediator sequence binds to the reporter and is extended by the polymerase, allowing the signal strength of the reporter to be evaluated.

Accompanying the separable optimization of the PCR sub-processes, mediator probe technology is characterized by a unique feature: optimized reporter structures can be transferred to new target DNA sequences, and only the DNA detection step needs to be developed for a new assay. The signal generation requires no further optimization [17]. Furthermore, high specificity towards single-nucleotide polymorphism (SNP) detection is achieved: during PCR primer extension, polymerases with exonuclease activity recognize the non-hybridized mediator sequences and cleave them off base specifically from the probe at its 5′-end [24]. Therefore, in the case that this 5′ end is positioned at an SNP, the mediator length will increase by one base if the mediator probe was not specific to this SNP. Consequently, a mismatch will be generated at the 3′ end of the mediator hybridized to the reporter, which will prevent mediator extension and thus fluorescence signal generation. As a result, only mediator probes specific to the respective SNP positions are capable of generating signals [20].

MP PCR has already proven useful in many assay developments. What it still lacks in a multiplex dPCR environment, however, is standardization of the reporter set development process. Systematically designed sets of reporters that can be used in different assays detecting multiple target panels are highly desirable for improved diagnostics of different genetic cancer markers in particular.

Our primary goal in this publication was to demonstrate how to systematically establish generic reporter sets for detection of exchangeable DNA target panels in multiplex dPCR assays as shown in Figure 1B. Therefore, at first, an optimized generic reporter set (hereafter referred to as the first generic reporter set) was characterized for colorimetric multiplexing (i.e., detecting one target per fluorescence channel), consisting of six reporters with different fluorophores and quenchers covering all six fluorescence detection channels of the naica^®^ dPCR system, a state-of-the-art dPCR platform. The detection and quantification capabilities of the first generic reporter set in combination with variable sets of DNA targets were investigated. These DNA targets consisted of multiple *KRAS*, *BRAF* and *NRAS* SNPs, which are commonly used as cancer markers for the detection and characterization of various cancer types [20,25,26,27,28], for example, in liquid biopsies [29,30,31]. However, DNA targets for other applications such as food testing have already been shown using MP PCR technology [17].

After characterizing the first generic reporter set, another novel and yet-to-be-optimized generic reporter set (hereafter referred to as the second generic reporter set), also for detection of 6-plex DNA target panels, was systematically optimized, in order to establish a generic reporter set comparable in performance to the previously characterized one. The purpose was to enable researchers to develop their own n-plex generic reporter sets for their individual needs. To this end, we describe in detail our generic reporter set development process and provide suggestions and recommendations for a simplified and standardized generic reporter set development and optimization workflow. In this context, we also provide MEA data on the fluorescence intensities and crosstalk behavior of 30 different fluorophores. These fluorophore data facilitate the evaluation of their potential compatibility with each other in a multiplexing environment, including the potential need to configure an appropriate color compensation, allowing faster development of novel generic reporter sets for other degrees of multiplexing. These other multiplexing degrees are not limited to colorimetric multiplexing techniques; the aforementioned higher-order multiplexing techniques may also be simplified and standardized using our guidelines.

Furthermore, we aimed to investigate the effects not only for variety of targets while using the same generic reporter set but also vice versa, with a variety of reporters while using the same targets and mediator probes. This approach may be used, for example, to rearrange generic reporter sets for other device platforms whose fluorescence channels have different specifications and therefore require different fluorophores.

## 2. Results

### 2.1. Characterization of a Fluorogenic Generic Reporter Set for Quantification of 6-Plex SNP Detection Panels

In this work, the focus was on how to systematically optimize generic reporter sets that can be used in combination with different target panels. For this purpose, this section addresses the characterization of an already optimized, first generic reporter set. In the following section, a systematic workflow for establishing a similarly well-performing, novel second generic reporter set from scratch is presented. The exact composition of both generic reporter sets, including sequences, fluorophores, quenchers and other properties, can be found in the supporting information (Tables S1 and S2).

First, it was examined whether all targets could be accurately detected and distinguished from each other. Therefore, the complete multiplex dPCR system, comprising all reporters as well as primers and mediator probes matching the target sequences, was tested in reactions with either one added DNA target sequence each or no template controls (NTC) with no DNA target and reactions in which all target sequences were present simultaneously at high concentrations of 10,000 copies per reaction (cpr). Two different target panels were used for this purpose: the first detected *KRAS* and *BRAF* wild-type (WT) alleles and corresponding SNPs, and the second target panel detected *KRAS* and *NRAS* WT alleles and corresponding SNPs. The two target panels had three identical targets (*KRAS* WT, G12A and G12D) and three targets differing from each other. Neither target panel was specifically optimized for use in combination with the first generic reporter set. Chemical compositions, such as oligonucleotide sequences and concentrations of the individual components, were taken from previous works [32] without adjustments, or standard concentrations were used, respectively. The PCR thermocycling profiles were also not adapted to the different targets.

**Figure 2:**
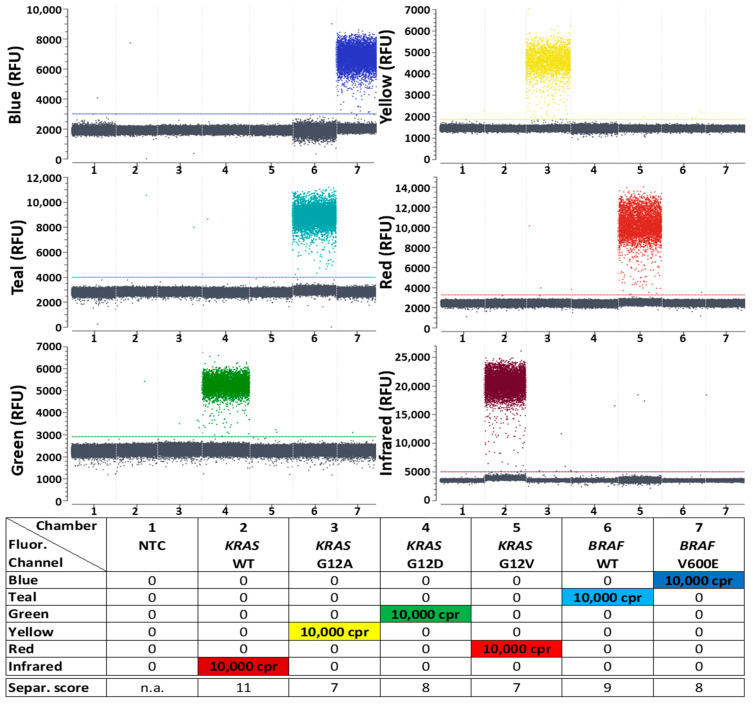
Differentiation between positive and negative signal populations in Digital PCR (dPCR) reactions using the first 6-plex generic reporter set in combination with a target panel for detection of *KRAS* and *BRAF* mutations and their corresponding WT controls. One-dimensional plots of all 6 fluorescence channels (labeled “Blue”, Teal”, “Green”, “Yellow”, “Red” and “Infrared”) of the naica^®^ Prism6 are shown. Details of the reporters used in each detection channel, including sequences, fluorophores and quenchers, can be seen in Table S1. All detectable droplets formed in seven separate reaction chambers are shown on the x-axis, and the relative fluorescence units (RFU) of each droplet are shown on the y-axis. Reaction chamber details from left to right: (1) no template control (NTC) without target molecule presence; (2–7) 10,000 copies per reaction (cpr) of one kind of target sequence each. A more detailed version of this figure, with samples containing all six targets at once, is shown in Figure S1. Separability scores between positive and negative droplet populations, as calculated by the Crystal Miner software, are indicated at the bottom of the legend. n.a.: not applicable.

Clearly distinguishable populations were formed in all fluorescence detection channels with the *KRAS* and *BRAF* target panel, as can be seen in 1D plots of the reactions containing one DNA target each (Figure 2). In the droplets of the lower populations, there was no target molecule present, whereas at least one target molecule was present in the droplets of the upper population. All NTCs showed negative results, meaning that the target concentration calculated by the Crystal Miner software version 4.0.10.3 was not within the 95% confidence interval of a positive result. This usually corresponded to fewer than four positive droplets per reaction, dependent on the total number of analyzable droplets in the reaction. Additionally, in all reactions with one target present, the respective fluorescence channel made it possible to differentiate between positive and negative droplet populations, whereas all other fluorescence channels for the detection of the absent targets failed to show positive populations. The clearly visible distinctiveness between positive and negative populations was confirmed by the separability scores, which were determined using Crystal Miner analysis software. In general, separability scores are based on the expansion of fluorescence intensities within populations and fluorescence intensity distances between populations [33]. The higher the separability score, the higher the distance between populations, suggesting better distinguishability from each other. Although the separability scores in the reaction with all six targets combined in high concentrations were expectedly lower than in the reactions with single targets, it was still possible to distinguish all populations (Figure S1).

**Figure 3:**
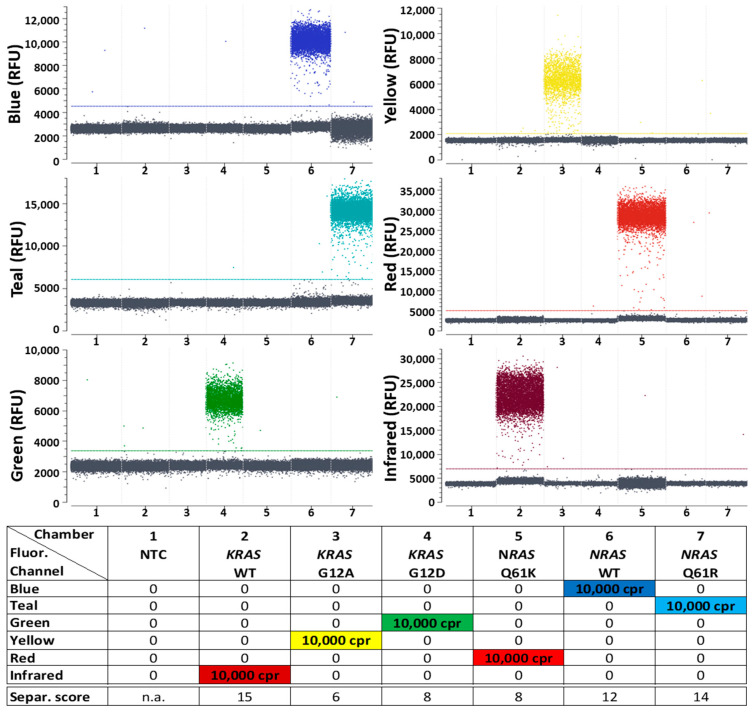
Differentiation between positive and negative signal populations in dPCR reactions using the first 6-plex generic reporter set in combination with a target panel for detection of *KRAS* and *NRAS* mutations and their corresponding WT controls. One-dimensional plots of all 6 fluorescence channels are shown. Details of the reporters used in each detection channel, including sequences, fluorophores and quenchers, can be seen in Table S1. Reaction chamber details from left to right: (1) NTC; (2–7) 10,000 cpr of one kind of target sequence each. A more detailed version of this figure, with samples containing all six targets at once, is shown in Figure S2. Separability scores between positive and negative droplet populations are indicated at the bottom of the legend.

The first generic reporter set in combination with the alternative target panel for the detection of *KRAS* and *NRAS* WTs and corresponding mutations enabled a similarly good population differentiability (Figure 3). The separability scores obtained with both target panels were not identical, but in a similar range. The detection of individual targets was possible in all cases by very well distinguishable droplet populations. A separation of the populations in samples with all targets simultaneously was also possible (Figure S2).

After checking the separability of all populations, the next objective was to evaluate the quantification capabilities of the first generic reporter set in combination with both target panels. For this purpose, 10-fold dilutions of the mutation targets were quantified on a constant background of WT targets. The lowest concentration tested (0.4 cp/µL) is close to the statistical detection limit of the naica^®^ system, which generates about 25,000 droplets per reaction with a volume of 0.68 nL per droplet [34], corresponding to about 17 µL of analyzed reaction mix. At a target concentration of 0.4 cp/µL, this corresponds to about six to seven target-containing droplets per reaction. These low concentrations lead to high statistically possible concentration fluctuations. If even lower target concentrations were tested, there might have been fewer than four target-containing droplets in the sample, which would then no longer correspond to a positive sample within the 95% confidence interval.

Liquid biopsy samples containing mutant DNA always also contain a significantly higher background concentration of WT DNA, resulting in low variant allele frequencies (VAF). To address this, high concentrations (400 cp/µL) of both WT targets were added to the samples, equaling a VAF of 0.1%, in order to simulate clinically relevant conditions [35] and to check whether the mutants can be quantified precisely in this more complex milieu.

**Figure 4:**
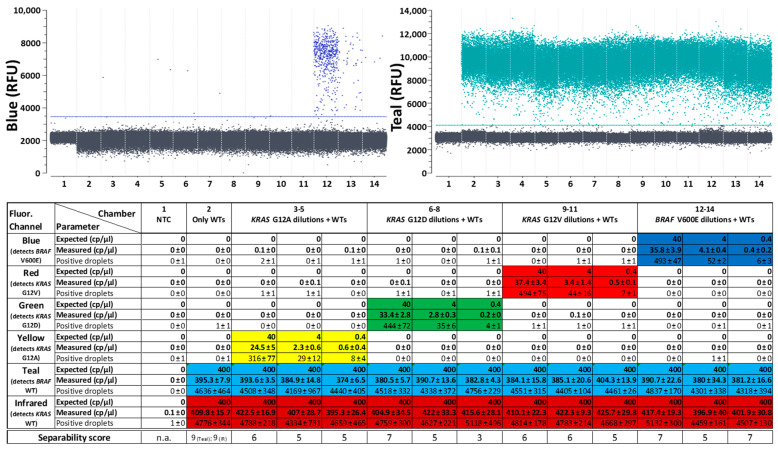
Quantitative dPCR results of the first 6-plex generic reporter set in combination with a target panel for detection of *KRAS* and *BRAF* mutations and their corresponding WT controls. A background of 10,000 cpr of both WT targets is present in all reaction chambers aside from the NTC (reaction chamber 1). Single mutation targets were added in three different concentrations (1000 cpr, 100 cpr and 10 cpr). Above: One-dimensional plots of fluorescence channels detecting either *BRAF* WT in “Teal” or *BRAF* V600E in “Blue”. One-dimensional plots of all other fluorescence channels detecting the other targets are depicted in Figure S6. Below: Quantitative data of three replicates with reactions freshly prepared for each experiment. Expected concentrations, mean values and standard deviations of measured concentrations in copies per microliter (cp/μL) as well as mean values and standard deviations of the number of positive droplets in all three experiments are shown for each reaction chamber in each detection channel. Cells are color-coded wherever a positive result was expected for the specific chamber and channel. Separability scores between positive and negative droplet populations in the depicted reactions are indicated at the bottom.

With the target panel for the detection of *KRAS* and *BRAF* targets, all corresponding mutants could be quantified in all tested concentrations (0.4–40 cp/µL) (Figure 4). All WTs could also be quantified well, despite the high concentrations used. Triplicate tests were carried out, with reactions freshly prepared for each individual experiment. The quantitative results obtained in all three separate tests are highly comparable, which shows that the quantification is reproducible and the standard deviations are low. At the lowest concentration levels measured, at least four positive droplets were seen in all performed tests. The samples were therefore always positive within a 95% confidence interval, even if the standard deviation in some cases (e.g., chamber 14 in the Blue channel) suggests that, under the conditions tested, it is in principle also possible to find only three positive droplets in a reaction chamber.

**Figure 5:**
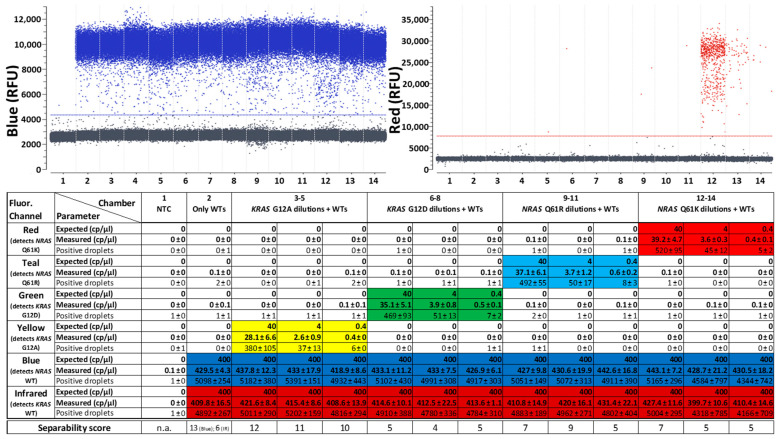
Quantitative PCR results of the first 6-plex generic reporter set in combination with a target panel for detection of *KRAS* and *NRAS* mutations and their corresponding WT controls. A background of 10,000 cpr of both WT targets is present in all reaction chambers aside from the NTC (reaction chamber 1). Single mutation targets were added in three different concentrations (1000 cpr, 100 cpr and 10 cpr). Above: One-dimensional plots of fluorescence channels detecting either *NRAS* WT in “Blue” or *NRAS* Q61K in “Red”. One-dimensional plots of all other fluorescence channels detecting the other targets are depicted in Figure S7. Below: Quantitative data of three replicates with reactions freshly prepared for each experiment. Expected concentrations, mean values and standard deviations of measured concentrations in cp/µL as well as mean values and standard deviations of the number of positive droplets in all three experiments are shown for each reaction chamber in each detection channel. Cells are color-coded wherever a positive result was expected for the specific chamber and channel. Separability scores between positive and negative droplet populations in the depicted reactions are indicated at the bottom.

Comparably accurate results were achieved with the target panel for the detection of *KRAS* and *BRAF* (Figure 5). Although the WTs of this target panel were partly located in different fluorescence channels than in the other target panel, the quantification of the WTs was similarly reliable. The quantitative tests provided reproducible results that corresponded closely with the expected concentrations and the mutations could still be detected close to the statistical detection limit. Reactions with the lowest concentrations measured (0.4 cp/µL) contained at least four positive droplets in all triplicate reactions, which means that these samples can all be considered to contain the target mutation within the 95% confidence interval.

In summary, it can be stated that the first generic reporter set reliably distinguishes all mutations and WTs of both tested target panels from each other. Regarding sensitivity, it can detect even a few molecules per reaction and can quantify them with a high accuracy down to 0.1% VAF. The steps required to establish and optimize such a generic reporter set are described in the following section using an alternative, second 6-plex generic reporter set, which was also developed as part of this study.

### 2.2. Development of a New Generic Reporter Set

In order to establish new generic reporter sets, fluorophores that are potentially compatible with the used device platform must first be identified. This can be achieved by first cross-checking device specifications with potentially suitable fluorophores and then carrying out MEAs with reporters that contain the selected fluorophores. MEAs allow the evaluation of fluorescence signal intensities for the tested fluorophore, independent of a complete PCR system including primers and mediator probes [19]. For this work, 30 different reporter-bound fluorophores were tested for the first time in a dPCR instrument via MEAs. The corresponding fluorescence intensities and fluorescence crosstalk with different color channels are shown in Figure S3, and the respective NTCs without mediators added to the reactions are shown in Figure S4. When analyzing this data in order to find a suitable generic reporter set, it is important to note that the MEA data shown is only valid if the same device platform type is used, as most dPCR platforms use different fluorescence detectors. It should also be noted that the fluorescence intensities generated in MEAs represent maximum values that are usually not achieved in PCR reactions containing the same reporter concentration, as target detection by primers and mediator probes usually does not reach a performance of 100% and consequently diminishes the efficiency of the subsequent signal generation step. However, the intensities shown can serve as a guidance as to whether a particular fluorophore is strongly or weakly fluorescing in the dPCR setting. In addition, the ratio of irradiation into different fluorescence channels in order to assess the expected crosstalk can be evaluated with the assistance of MEAs, as this ratio typically stays the same in MEAs and PCRs.

**Figure 6:**
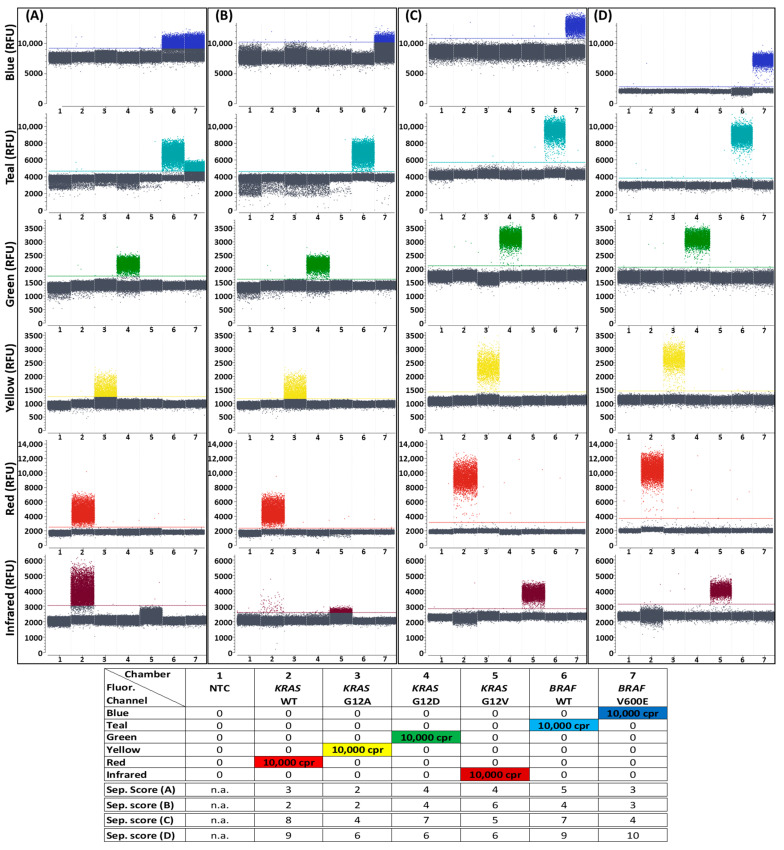
Improving discriminability between positive and negative signal populations in dPCR reactions step-by-step. During all steps of generic reporter set optimization, the target panel for detection of *KRAS* and *BRAF* mutations and their corresponding WT controls was used. 1 D-plots of all 6 fluorescence channels are shown. Reaction chamber details from left to right: (1) NTC; (2–7) 10,000 cpr of one kind of target sequence each. A more detailed version of this figure, with samples containing all six targets at once, is shown in Figure S5. Separability scores between positive and negative droplet populations are indicated at the bottom of the legend. Fluorophores for this second generic reporter set were selected according to the MEA data shown in Figure S3. Details of the used reporters in each detection channel including sequences, fluorophores and quenchers can be seen in Table S2. (**A**) Initial non-optimized test without color compensation. (**B**) Initial non-optimized test after setting a suitable color compensation, with which each target is detected in only one fluorescence channel. This color compensation matrix was henceforth used in every subsequent test to optimize the second generic reporter set. (**C**) Improved separability after increasing the PCR cycle number from 45 to 60. (**D**) Further improved separability after reduction of background fluorescence in the “Blue” and “Teal” channels by changing the naica^®^ master mix version from “naica^®^ multiplex PCR MIX” to “naica^®^ PCR MIX”. Although the second generic reporter set shown here is an optimized set, ready to be used in combination with other target panels, it utilizes different reporters from the first 6-plex generic reporter set that was characterized in the *KRAS*, *BRAF* and *NRAS* quantification studies shown in Section 2.1.

The data from Figure S3 was used for the second generic reporter set shown below (Figure 6), just as for the first generic reporter set that was already characterized in the previous section, and can also be consulted for any further generic reporter set development on the naica^®^ Prism6 platform. The fluorophores used for both sets can be found in Tables S1 and S2. No fluorophores that emit only in the Blue or only in the Red detection channel could be identified; therefore, crosstalk with other channels which could afterwards be eliminated by color compensation was accepted when selecting all the fluorophores for the second generic reporter set. Important optimization steps that led to the dPCR results shown in Figure 6D (with analogous experimental setup and sample numeration to Figure 2) are explained below:

First, the selected fluorophores coupled to reporters were combined to a generic reporter set and tested in combination with the target panel for the detection of *KRAS* and *BRAF* mutations and corresponding WTs in a dPCR (Figure 6A) under standard conditions (Table 1 and Table 2). The resulting 1D plots show that the positive and negative droplet populations can only barely (Teal, Green and Red) or not at all (Blue, Yellow and Infrared) be distinguished from each other, and the corresponding separability scores confirm insufficient discriminability. In addition, there was strong crosstalk between the signals generated for the Blue and Teal channels and the signal intended for the Red channel emitted even stronger in the Infrared channel than the other fluorophore that was intended for this channel.

The most serious issue of misdetection of targets in other channels due to crosstalk could be solved by setting a suitable color compensation (Figure 6B). The applied color compensation matrix was generated using the Crystal Miner function “Compute Spillover Compensation”, assigning the reactions containing single targets (reactions 2–7) to the fluorescence channels intended for the detection of these targets. The color compensation only prevented target detection in the wrong channels, but did not improve the separability of the positive from the negative droplet populations. The color compensation also led to undesirable effects of the baselines due to the generally poor separability of the populations from each other, which was mainly expressed by broadened baseline populations in the Teal but also in the Blue channel.

By increasing the number of dPCR cycles from 45 to 60, the discriminability of the negative from the positive droplet populations was visibly improved in all detection channel 1D plots, which is also reflected in the increased separability scores in almost every channel (Figure 6C). Due to the improved population separability, the color compensation was also able to generate more precise baselines consisting of more densely packed negative droplet populations in the Teal channel.

A further improvement in the distinguishability between negative and positive droplets, especially in the Blue and Teal channels but also to a lesser extent in other channels, was achieved by adjusting the background fluorophore (Figure 6D). Background fluorescence in the Blue channel is necessary to detect droplet positions in the reaction chambers for subsequent data analysis. In the previous experiments, a standard master mix for the naica^®^ system was used, which already contains a background fluorophore emitting in the Blue and Teal channels to localize the droplets for subsequent analysis. However, this background fluorophore strongly increased the fluorescence intensity in the Blue and Teal channels, making it more difficult to detect positive droplets in these channels. Since the second generic reporter set already contained two fluorophores (FAM and Atto488) that fluoresce in the wavelength ranges required for localizing the droplets and thus, even when quenched, generate sufficiently strong background fluorescence, the background fluorophore was not required in this assay. Therefore, a different master mix was selected for this experiment, which differs from the previously used master mix only in the absence of the background fluorophore. This adjustment concluded the assay optimization performed for the second generic reporter set.

The optimization increased not only the distinctness of the populations in the presence of individual targets but also the distinctness in the presence of multiple targets (see sample number 8 and corresponding separability scores in Figure S5). Although, as expected, the separability of all targets in the same reaction is lower at the applied high concentrations than when detecting individual targets, at the end of the optimization all targets were not only simultaneously detectable but also provided quantitative results that corresponded to those obtained with the individual targets, indicating a functional population separability despite lower separability scores.

## 3. Discussion

### 3.1. Impact of the Developed Generic Reporter Sets

In this work, a robust and already optimized first 6-plex generic reporter set, which allows high fluorescence population separation in colorimetric digital multiplex PCR and precise, sensitive and specific DNA quantification for different target panels, was characterized. This characterization was achieved by the specific quantification of two different target panels in Section 2.1. The first generic reporter set showed high reproducibility of results with both tested target panels. Since target detection and signal generation are decoupled in MP PCR, target panels are interchangeable as already shown exemplarily in this work. This results in following advantages when using universal reporter oligonucleotides in combination with mediator probes for improving dPCR assays:

- Efficient oligonucleotide design: One generic reporter set can be used for different colorimetric multiplex assays. When using the same dPCR platform, no further optimization steps are needed. For other platforms, generic reporter set optimization is necessary only once.
- Higher degree of freedom and deeper insight in assay design: The differentiability and quantifiability of the respective targets used is independent of the fluorescence signal generation, since signal generation is standardized by the generic reporter set. Alternative, TaqMan^TM^-based approaches exhibit a higher dependency of fluorescence signal generation on the respective target-specific probe sequence. For example, guanine bases are known to quench the fluorescence of several fluorescent dyes when in close proximity [36,37]. If this type of quenching is attempted to be avoided by changing the length of the probe, shortening the probe often makes it less specific and extending it reduces the efficiency of the quencher, which is usually located at the other end of the TaqMan^TM^ probe [38]. When using generic reporter sets instead, if individual targets do not yield proper fluorescence signals, the problem lies not in the generic signal generation but in the decoupled target detection, which is why fewer parameters need to be checked during troubleshooting.
- Good absolute quantifiability: Quantification results show little difference between expected and measured copy numbers, as has already been shown by Schlenker et al. [20]. dPCR in general has a better quantitative resolution than standard qPCR [39] which is resolved via comparing threshold cycle numbers that can be dependent on many factors as inhibitors [40,41] and the applied detection system [42]. With the presented assay, SNP mutations can still be reliably quantified in presence of at least 1000-fold excess of the corresponding WT (plus another WT) in the same reaction. It can be assumed that even higher WT excesses do not present a problem for mutation target detection, since the detection of mutations in the presence or absence of the highly concentrated WTs did not show any noticeable differences, while many of the mutation target-positive droplets must already have contained WT targets when adding them in 1000-fold concentrations.
- Very high sensitivity: As few as four copies (four positive droplets) per compartmented reaction volume in positive samples could be detected with the naica^®^ dPCR system, which is more sensitive than, for example, common qPCR assays [43,44].
- High specificity: Despite the simultaneous detection of different SNPs, the assays detected the correct targets, differentiating reliably between single base mismatches [20]. In all tested reactions, the number of false positives was so low that it was never within the 95% confidence interval for a positive result.

As a limitation of our study design, we did not attempt quantification on a physiological background of fragmented DNA, as is present, for example, in real liquid biopsy samples. However, in an earlier work, Schlenker et al. did prove applicability of the used MP PCR technology for quantification in plasma samples of colorectal cancer patients [20]. Reporter sequences and modifications including fluorophores and quenchers can be found in Tables S1 and S2. Information on the mediator probes and primer sequences that were used for the analyzed targets is given in Table S3. Sequence, modification layout and fluorophore-quencher configuration are important reporter parameters since fluorogenic properties are dependent on an optimized minimum distance between fluorophore and quencher, which can only be achieved via suitable sequence motifs [19]. Further information on oligonucleotide design for mediator-probe-based assays can be found in [17,23], among other publications.

**Figure 7:**
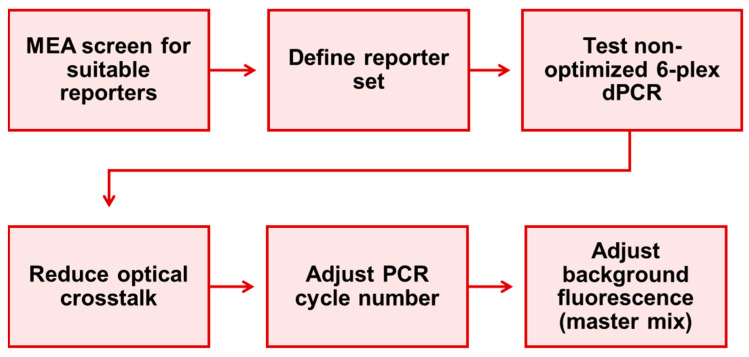
6-plex dPCR assay optimization workflow for definition of a generic reporter set as performed in this work and recommended for the establishment of new generic reporter sets.

In addition to this first generic reporter set, a second 6-plex generic reporter set for colorimetric multiplexing was developed in this work in order to demonstrate the applicability of the general signal optimization workflow for creation of new generic reporter sets as discussed later and shown in Figure 7. In contrast to the first generic reporter set in Section 2.1, the second generic reporter set was not characterized regarding its quantification performance. It was instead characterized regarding fluorogenic properties in consecutive steps of digital PCR assay development as guidance for the design of new generic reporter sets that will be needed for example when using different dPCR devices or degrees of multiplexing.

Critical examination of the two target panels tested with the first generic reporter set reveals that there are more or less pronounced fluorescence intensity differences in several detection channels depending on the used targets. The most obvious difference was seen in the Red channel. They do not affect the quantification and distinguishability of the populations and might be caused by different binding strengths between oligonucleotides that are used as parts of the respective target panels, depending on the nucleotide sequences. Another cause might be unspecific interactions between oligonucleotides. However, no obvious binding strength differences between tested panels or unspecific interactions were observed in silico with OligoPAD software version 0.3.9.4 (see Section 4.1). Another fact to consider is that the tested target panels included many SNPs that differed from each other in only one single base, for the detection of which the same primers and the same binding sites of the mediator probes to the respective targets were used. In a regular multiplexing approach without using MP technology, such constellations could lead to the individual assays impairing each other’s performance. However, as already shown in earlier publications [20,32], one of the strengths of MP technology lies in its ability to distinguish SNPs from one another, which is also evident in the sequences tested in this work.

Another apparent irregularity is that the MEA results in some channels show lower fluorescence intensities than the positive populations of the PCR results in Figure 2, Figure 3 and Figure 6D. This seems to be a contradiction, as in MEAs the maximum possible fluorescence intensity of a reporter is reached by direct activation of the reporter by means of free mediators. A simple explanation for this is that the reporter concentration in the MEAs was four times lower than in the PCRs (see Table 1 and Table 3), because otherwise some fluorophores in the MEA would have surpassed the upper fluorescence detection limit of the dPCR instrument.

### 3.2. Strategies to Reduce Signal Crosstalk

In the experiments performed for Figure 4 and Figure S6, respectively, the Infrared channel shows a slightly more upwardly scattering baseline population, which could also be a secondary population directly above the baseline in chamber 9. This is due to fluorescence crosstalk from the Red channel fluorophore Atto 647N, since the Red channel target is also present in the same sample. This crosstalk is actually supposed to be avoided by the color compensation, which does occur in the vast majority of samples. However, minor over- or under-compensation cannot be ruled out. False positive detection of mutations is far more critical than false positive WTs, that are expected in each sample under physiological conditions anyway. It is therefore important to set the color compensation or assign the targets to detection channels in such a way that, in the event of a slight crosstalk, the scattered baseline population does not negatively affect the separability of mutation populations. This fact was taken into account when fine-tuning the color compensation of both target panels: From the MEA data (Figure S3) it could be concluded that the fluorophores used for the first generic reporter set would mainly cause fluorescence crosstalk between the Blue and Teal channel (utilizing FAM and Atto488 fluorophores) as well as between the Red and Infrared channel (with Atto647N and Cy5.5), which needed to be compensated. Since the Red channel fluorophore Atto647N fluoresces much more strongly into the Infrared channel than the Infrared fluorophore Cy5.5 fluoresces into the Red channel, the target with the *KRAS* G12V mutation was assigned to the Red channel and the corresponding WT to the Infrared channel in the *KRAS* + *BRAF* target panel (see Figure 2 and Figure 4). Another strategy for addressing fluorescence crosstalk irregularities was demonstrated in the Blue and Teal channels with the same target panel. Therein, the Atto488 fluorophore used for the Teal channel has stronger crosstalk with the Blue channel than the Blue channel fluorophore FAM has with the Teal channel. Nonetheless, the *BRAF* WT could be quantified in the Teal channel and the corresponding mutant *BRAF* V600E in the Blue channel. Therefore, the color compensation was adjusted in a way that crosstalk from Teal to Blue was compensated rather a little too strongly than too weakly, which resulted in a Blue channel baseline that was broadened downwards and consequently was not critical for the separability of the mutation from other populations (see Figure 4, Blue channel, baselines of samples 2–14).

The necessity to minimize signal crosstalk can in principle also be circumvented by replacing individual fluorophores with other ones that have more favorable fluorescence properties for the multiplex assay, for example, by not having crosstalk with other detection channels. However, such fluorophores that are detected in only one particular channel are often not available as can be seen for the Blue and Red channels in Figure S3. Furthermore, even if it was possible to identify respective fluorophores for all detection channels, they are not automatically a reasonable component of every subsequent reporter set design. This is because most device platforms use different fluorescence detectors and filters with their respective wavelength optima and limits, making different fluorophores a good choice for different devices. In addition, changes in assay composition, such as altered pH [45] or viscosity due to master mix changes, affect fluorophore quantum yields and consequently influence fluorescence intensities [46], which can reduce the applicability of fluorophores.

### 3.3. Parameters for Evaluation of dPCR Result Quality

Density plots, separability scores and similar overviews or algorithms that are used to evaluate and compare the discriminatory power of data points inside clusters or between different clusters are a useful tool in dPCR. Separability scores are calculated based on distances between populations (data point clusters) and the spread of data points within the examined populations [33]. The higher the scores, the better the relative separability between populations. This scoring system can be used to evaluate the influence of rain on the data analyzability. It is, for example, also used when different protocols for a singleplex dPCR assay are compared regarding differentiability between populations when the target population fluoresces more intensively but the data points within the population are also more broadly scattered. Separability scores however often fail, when the distinctiveness of more than two populations needs to be evaluated simultaneously, as for example in higher order multiplexing approaches or in the presence of undesired co-amplification populations, since the scores always only compare two populations with each other.

During the generic reporter set optimizations carried out for this work, it was noticed in some cases that the separability of the populations clearly improved from the 1D plots, but the separability score did not improve at the same time or even decreased slightly. Examples that can be seen in Figure 6 are the changes between detection of *BRAF* WT in (A) and (B) and of *KRAS* G12V in (B) and (C). In the first case, the separability score was slightly reduced in (B), although there was no longer any false detection of the *BRAF* WT in the Blue channel, which must be regarded as a clear improvement. In the second case, the separability score was reduced, although the detectability of the target in the Infrared channel was strongly improved. Another example with a different cause can be found in Figure 4 and Figure S6, respectively: chamber 8 in the Green channel. The very low separability score of 3 therein is not due to a low separability of the positive population from the baseline population, but is mainly due to the low number of positive droplets. These cases show that the separability scores not always reflect the quality of results and that the automated evaluation of dPCR experiments still needs to be checked manually. Improved separability score algorithms or alternative parameters for categorizing the discriminatory power between populations may provide assistance for a better evaluation.

Especially for analysis of quantitative experiments in the context of this work, the 95% confidence interval has proven to be a reliable and useful measure. In rare instances, either due to contaminations or due to unpredictable or unknown side reactions, individual droplets in a batch may lead to an increase in signal without the presence of a corresponding target. False positive results or a reduction in assay specificity are the consequence if confidence intervals are not included in the result evaluation. The 95% confidence interval suggests that, depending on the exact number of droplets generated and analyzable, the reaction may contain a small number of positive outliers without the sample being counted as positive. Conversely, however, this also means that the ability to detect only a single target molecule per sample, for which dPCR is sometimes praised [47,48], is ruled out if the 95% confidence interval is taken into account. In other words, the lower bound of the 95% confidence interval for the concentration of a sample containing only one target molecule usually has a value below 0. The maximum sensitivity specified by the 95% confidence interval in the case of approx. 25,000 generated droplets per Sapphire chip reaction chamber is four copies per reaction. This basic assumption was taken into account for quantitative analysis of the experiments performed in this study. Our results show that all samples with the lowest tested concentration of 0.4 cp/µL target DNA contained at least four positive droplets and were consequently evaluated as positive for the target. In addition, all samples without added target contained less than four positive droplets and were therefore correctly classified as negative. Especially for the early detection or minimal residual disease of tumors in a liquid biopsy, this clear definition can be useful to prevent false positive results while still maintaining a high sensitivity. However, it is important to consider that our observed limit of four positive droplets is a variable value that depends, for example, on the device platform specifications including the number of compartments per reaction. The 95% confidence interval is therefore the decisive parameter when calculating the actual number of positive droplets required to label a sample as positive or negative for a specific target.

Another parameter for assessing fluorescence signal intensity in the context of PCR developments is the signal-to-noise ratio (SNR) [19,49,50,51], which focuses less on the scattering within clusters or populations. This parameter is mainly used in qPCR-based methods and can also be understood as the qPCR equivalent to the separability scores in dPCR.

### 3.4. Workflow for the Generic Creation of New Reporter Sets

In the following, the workflow (Figure 7) is described, which was used to establish the generic reporter sets developed as part of this work. This workflow represents the step-by-step experimental procedure necessary to carry out the general design principle shown in Figure 1B in a standardized way. The workflow thus enabled not only the development of the first generic reporter set (see Section 2.1) but also the development of the second generic reporter set described in Section 2.2 and can be used analogously to develop further generic reporter sets with other fluorescence properties or other multiplexing degrees by adjusting the reporter identity and number.

First, relevant fluorophores suitable for coupling to a reporter must be selected, corresponding reporters labeled with these fluorophores and a suitable quencher must be synthesized and all these reporters must be characterized in a MEA in order to analyze their excitation and emission properties in the available fluorescence channels. Since fluorophores that have already been characterized in a MEA do not need to be tested again for further generic reporter set developments on the same device platform, this step can be omitted in most cases when using the naica^®^ Prism6 in the future. Corresponding MEA data for 30 fluorophores distributed over the entire spectrum of the naica^®^ Prism6 detectors are already shown in Figure S3.

From the characterized reporters, candidates that can be used for the desired generic reporter set must be selected according to the desired number of reporters in the set and the desired wavelength ranges. The reporters are then tested together for the first time in a dPCR, whereby it is advised to use primers and mediator probes from an already established standard assay in order to avoid false negative results due to unfavorable primer and probe sequences. In most cases, this first test will result in at least some of the targets being detected in more than one fluorescence channel simultaneously. If a maximum of one target per detection channel is to be detected, this can be achieved by applying a straightforward, automated color compensation procedure. For higher order multiplexing with several reporters per detection channel, the color compensation matrix may need to be adjusted manually in order to enable clean detection of the targets without reduced separability of the populations due to fluorescence crosstalk. For highly multiplexed generic reporter sets, it is generally favorable to select fluorophores which are only detected in one fluorescence channel if possible, as for example Cy3 in the Green channel (Figure S3) to avoid color compensation errors due to a high complexity of the compensation matrix. Further information on setting a suitable color compensation matrix can be found in Section 3.2.

In the next optimization step, the fluorescence intensity of the populations is adjusted by varying the number of dPCR cycles in further experiments until an optimum value is reached. The principle here is: The more cycles are run, the more reporters are activated in target-containing droplets and thus the higher the fluorescence intensity of the positive droplets is. Simultaneously, an increased number of cycles also increases the overall PCR duration and the probability of non-specific false detection and the fluorescence intensity of the negative droplet populations. However, since MP PCR is known for its high specificity, we never observed this in our experiments [20]. We therefore recommend to test high cycle numbers of approximately 60 in order to increase the overall signal strength, especially in highly multiplexed assays, where the PCR efficiency is naturally lower than in singleplex assays and tends to be unequally distributed between different targets when amplified in parallel [52,53]. A major reason for the lower loss of specificity when increasing the number of cycles in dPCRs compared to qPCRs is that spontaneously occurring side reactions, which lead to incorrect signal generation, can only affect digital MP PCR in a spatially limited area, namely in a single droplet, whereas in qPCR they can spread over the entire reaction volume.

In the final optimization step, the background fluorescence of the channel in which the droplets or compartments are detected is adjusted in further dPCR tests, depending on the used device platform. In case of the naica^®^ platform, it might be reasonable to replace the naica^®^ master mix containing a background fluorophore with the otherwise identical master mix without background fluorophore. If there is at least one reporter in the generic reporter set that emits in the channel used for droplet detection, no or at least a lower concentration of background fluorophore is required for droplet detection in the assay. This procedure can reduce the fluorescence intensity of the negative population in the corresponding fluorescence channel, which has a positive effect on the distinctness of the populations in this channel and may also result in improved population separability in other channels due to a facilitated color compensation.

We encourage to use the presented workflow for the development of new generic reporter sets with different degrees of multiplexing or for other dPCR platforms.

## 4. Materials and Methods

### 4.1. Reagents and Devices

Oligonucleotides were designed according to Lehnert et al. [19] using the software OligoPAD version 0.3.9.4 (Gesellschaft für naturwissenschaftliche Informatik mbH, Dortmund, Germany) if not derived from Calabrese et al. [32]. Template sequences were purchased as double-stranded gBlocks^TM^ Gene Fragments (Integrated DNA Technologies, Leuven, Belgium). Lyophilized template sequences were dissolved in 1 × TE buffer (10 mM Tris-HCl and 1 mM EDTA in nuclease-free water, pH: 7.6) to 10 ng/µL stock concentration according to manufacturer instructions and stored at −20 °C. All oligonucleotides including primers, mediators, mediator probes and reporters were synthesized and HPLC-purified from biomers.net GmbH (Ulm, Germany). Lyophilized oligonucleotides were dissolved in nuclease-free water to 100 μM stock concentration and stored at −20 °C. Oligonucleotide and synthetic template sequence information is provided in the supplementary information (Table S3). naica^®^ multiplex PCR MIX 10× and naica^®^ PCR MIX 10×, each including buffer A with a 10× stock concentration and buffer B with a 100% stock concentration, were purchased from Stilla Technologies (Villejuif, France). Herring sperm DNA (Catalog No. D181A, Promega GmbH, Walldorf, Germany) was diluted in nuclease-free water to a working stock concentration of 10 mg/mL.

Assays were performed in naica^®^ Geode cyclers (Stilla Technologies, Villejuif, France), using Sapphire chips (Stilla Technologies, Villejuif, France) for droplet generation.

### 4.2. Mediator Extension Assays and Digital PCR

dPCR master mix concentrations (Table 1) and cycling profiles (Table 2) initially oriented on Calabrese et al. [32] and were optimized step-by-step according to the information given in the results section. Primer concentrations inside master mixes depended on sequence identities with multiplex assays containing 2 µM *KRAS* forward primer and 1 µM *KRAS* reverse primer, 1 µM *BRAF* forward primer and 0.5 µM *BRAF* reverse primer and/or 1 µM *NRAS* forward primer and 2 µM *NRAS* reverse primer. Herring sperm DNA was used in the 10 mg/mL working stock concentration for diluting gBlocks^TM^ target DNA which resulted in a final reaction concentration of 2.4 mg/mL Herring sperm DNA.

**Table 1:** List of dPCR mix components, manufacturers and respective final concentrations in Sapphire chip reaction chambers. In mixes containing more than one reporter and mediator probe, the indicated concentration refers to the final concentration per oligonucleotide. Primer concentrations were as follows: 2 µM *KRAS* forward primer, 1 µM *KRAS* reverse primer, 1 µM *BRAF* forward primer and 0.5 µM *BRAF* reverse primer for 6-plex assays detecting *KRAS* and *BRAF* targets; 2 µM *KRAS* forward primer, 1 µM *KRAS* reverse primer, 1 µM *NRAS* forward primer and 2 µM *NRAS* reverse primer for 6-plex assays detecting *KRAS* and *NRAS* targets.

Component	Producer	Final Concentration
Buffer A—naica^®^ (multiplex) PCR MIX 10 X	Stilla Technologies	1×
Buffer B—naica^®^ (multiplex) PCR MIX	Stilla Technologies	4%
H_2_O	Qiagen (Hilden, Germany)	-
Reporter	biomers.net	0.4 µM
Mediator probe	biomers.net	1.2 µM
Primers	biomers.net	0.5–2 µM (depending on assay)
Target DNA	Integrated DNA Technologies	variable
Herring sperm DNA	Promega	2.4 µg/mL

The initially applied PCR cycling profile consisted of a denaturation step over 3 min at 95 °C followed by 45 amplification cycles with 15 s at 95 °C and 60 s at 58 °C. In further course of the experiments, the number of amplification cycles was increased to 60 for better signal generation as described in the results section, whereas the other conditions did not change. The initial partitioning and terminal pressure release steps are specific to the naica^®^ system and were carried out as recommended by the manufacturer.

**Table 2:** Final dPCR cycling profile with increased cycle number.

	Temperature [°C]	Duration [min:s]	Cycles
Partitioning	40	12:00	-
Initial denaturation	95	3:00	-
Amplification	95	0:15	60
	58	0:60	
Pressure release	25	33:00	-

MEA oligonucleotide concentrations (Table 3) were derived from Lehnert et al. [19] and master mix composition as well as the cycling profile (Table 4) were adapted for digital MEA applications. Most notably, the number of amplification cycles was decreased from 45 to 20 cycles because sufficient signal generation was already observed after low cycling numbers.

**Table 3:** List of MEA mix components, manufacturers and respective final concentrations in Sapphire chip reaction chambers.

Component	Producer	Final Concentration
Buffer A—naica^®^ multiplex PCR MIX 10 X	Stilla Technologies	1×
Buffer B—naica^®^ multiplex PCR MIX	Stilla Technologies	4%
H_2_O	Qiagen	-
Reporter	biomers.net	0.1 µM
Mediator	biomers.net	0.15 µM

**Table 4:** MEA cycling profile.

	Temperature [°C]	Duration [min:s]	Cycles
Partitioning	40	12:00	-
Initial denaturation	95	5:00	-
Amplification	95	0:10	20
	60	0:30	
Pressure release	25	33:00	-

### 4.3. Data Analysis

Scans of the cycled Sapphire chips were performed in a naica^®^ Prism6 scanner with following absorption wavelength exposure times: Blue channel: 100 ms; Teal channel: 350 ms; Green channel: 100 ms; Yellow channel: 125 ms; Red channel: 500 ms; Infrared channel: 500 ms. Data was analyzed with Crystal Miner software version 4.0.10.3 (Stilla Technologies, Villejuif, France). Spillover compensation was performed according to manufacturer recommendations in order to minimize crosstalk between the six fluorescence channels of the Prism6. Exclusion of irregularly shaped or unevenly colored droplets or Sapphire chip chamber areas was performed using the Crystal Miner quality control selection menu. A reaction was considered negative for a particular color channel, if the minimum target concentration calculated by the Crystal Miner software was below zero, indicating that the result was not within the 95% confidence interval of a positive result. The threshold for a sample to be positive was dependent on the total number of analyzable droplets in the Sapphire chip chamber and was typically reached when more than three positive droplets were detected in the chamber. Separability scores were determined using the “Auto” fluorescence threshold function of the Crystal Miner software.

Separability scores were calculated automatically in the naica^®^ platform by an algorithm for each sample and channel in order to evaluate the quality of the generated experimental data. Separability score calculation was based on the distance between populations (meaning clusters) and the spread inside the examined populations [33]. High scores indicated a high degree of separability between the populations while scores below four indicated only partial separability between populations [54].

## 5. Conclusions

In this work, we presented two generic reporter sets for 6-plex detection with the naica^®^ Prism6 using mediator probe technology, showed basic design principles for development of new generic reporter sets for more highly multiplexed dPCR assays and, in this context, provided MEA data for 30 reporters with different fluorophores. By providing this knowledge, we enable other researchers in the dPCR community to use our generic reporter sets in combination with their own targets or to create their own generic reporter sets using our guidelines in order to enable standardized high-plex assays for a multitude of different target panels.

## 6. Patents

MP PCR is protected by the patents/patent applications based on the patent family published under WO2013079307 (Bifunctional oligonucleotide probe for universal real-time multianalyte detection).

## Data Availability

Data is contained within the article and Supplementary Materials.

## Supplementary Materials

The following supporting information can be downloaded at: https://www.mdpi.com/article/10.3390/ijms25168968/s1.
