# Supplementary material for: Generic Reporter Sets for Colorimetric Multiplex dPCR Demonstrated with 6-Plex SNP Quantification Panels
Source: Int J Mol Sci. 2024 Aug 17;25(16):8968. doi: 10.3390/ijms25168968 (PMC11355019; doi:10.3390/ijms25168968)
Supplement: Supplementary file 1 [file ijms-25-08968-s001.zip › ijms-3083521-supplementary.pdf]

Table S1. Sequences, fluorophores, quenchers and other properties of the characterized generic reporter set.

PSR number	Detection channel	Sequence (5´->3´)	3'-modification	5´- modification	Internal modification
1	Blue	ATGCCGATTAGATGCGGCA8TCGATCACACAACATGAGCATGTGTAC	UR-Block (C3-Spacer)	BHQ-1	8=dT-FAM
2	Teal	ATCCGCCAAGACGCGCGGA8TAGCATGTGAGGAACACGATGACAC	UR-Block (C3-Spacer)	BMN-Q1	8=dT-Atto488
3	Green	GACCGGCTAAGACGCGCCGGT7TGTTGCACCTGGGACATCGACTAT	UR-Block (C3-Spacer)	BMN-Q-535	7=dC-BDP-TMR
4	Yellow	GACCGCACTAGTAGATGCGGT5TGTCGTGGACCCTGTATCGAGCA	UR-Block (C3-Spacer)	BMN-Q-535	5 = dC-Dy-590
5	Red	GACGCGTAGTACAGAACGCGT7TGTTCAGTGAGCCTACCTGCCTTC	UR-Block (C3-Spacer)	BHQ-2	7 = dC-Atto-647-N
6	Infra-red	GACCGGCCAAGACGCGCCGGT7TGTTCACTGACCGAACTGGAGCA	UR-Block (C3-Spacer)	BHQ2	7 = dC-Cy5.5

Table S2. Sequences, fluorophores, quenchers and other properties of the generic reporter set that was used in **Figure 6**.

PSR number	Detection channel	Sequence (5´->3´)	3'-modification	5´- modification	Internal modification
1	Blue	ATGCCGATTAGATGCGGCA8TCGATCACACAACATGAGCATGTGTAC	UR-Block (C3-Spacer)	BHQ-1	8=dT-FAM
2	Teal	ATCCGCCAAGACGCGCGGA8TAGCATGTGAGGAACACGATGACAC	UR-Block (C3-Spacer)	BMN-Q1	8=dT-Atto488
3	Green	GACCGGCTAAGACGCGCCGGT7TGTTGCACCTGGGACATCGACTAT	UR-Block (C3-Spacer)	BMN-Q-535	7=Cy3
4	Yellow	GACCGCACTAGTAGATGCGGT5TGTCGTGGACCCTGTATCGAGCA	UR-Block (C3-Spacer)	BHQ2	dC-Cy3.5
5	Red	GACCGGCCAAGACGCGCCGGT6TGTTCACTGACCGAACTGGAGCA	UR-Block (C3-Spacer)	BHQ2	6=dC-Dy636
6	Infra-red	GACGCGTAGTACAGAACGCGT2TGTTCAGTGAGCCTACCTGCCTTC	UR-Block (C3-Spacer)	BHQ2	2 = Atto680

Table S3. Oligonucleotide and synthetic template sequences used for dPCR experiments.

Oligo type	Target	Sequence (5´->3´)	3'-modification
Mediator probe	*KRAS* WT	CTCCAGTTCGGTCCAGCTCCAACTACCACAAGTTTATATTCAG	MP-Block (C3-Spacer)
	KRAS G12A	GATACAGGGTCCACTGGCGTAGGCAAGAGTGCCTTGACG	MP-Block (C3-Spacer)
	KRAS G12D	ATGTCCCAGGTGCATGGCGTAGGCAAGAGTGCCTTGACGAT	MP-Block (C3-Spacer)
	KRAS G12V	AGGTAGGCTCACTTGGCGTAGGCAAGAGTGCCTTGACGAT	MP-Block (C3-Spacer)
	BRAF V600E	ACATGCTCATGTTGTGTCTGTAGCTAGACCAAAATCACCTATTTTTACTGTGAG	MP-Block (C3-Spacer)
	BRAF WT	GTGTTCCTCACATGCTACTGTAGCTAGACCAAAATCACCTATTTTTACTGTGAG	MP-Block (C3-Spacer)
	NRAS Q61K	GCAGGTAGGCTCACTTCCAGCTGTATCCAGTATGTCCAACAAACA	MP-Block (C3-Spacer)
	NRAS WT	ATGCTCATGTTGTGTGATGTCCAGCTGTATCCAGTATGTCCAACAAA	MP-Block (C3-Spacer)
	NRAS Q61R	CGTGTTCCTCACATGCGTCCAGCTGTATCCAGTATGTCCAACA	MP-Block (C3-Spacer)
Primer	*KRAS* (forward)	GGCCTGCTGAAAATGACT	-
	KRAS (reverse)	ACAAAATGATTCTGAATTAGCTGTA	-
	BRAF (forward)	GACCCACTCCATCGAGATTTC	-
	BRAF (reverse)	GCTTGCTCTGATAGGAAAATGAG	-
	NRAS (forward)	CTCTCATGGCACTGTACTCTTC	-
	NRAS (reverse)	AACAAGTGGTTATAGATGGTGAAAC	-
gBlock DNA Target	*KRAS* WT	CATTTTCATTATTTTTATTATAAGGCCTGCTGAAAATGACTGAATATAAACTTGTGG TAGTTGGAGCTGGTGGCGTAGGCAAGAGTGCCTTGACGATACAGCTAATTCAGAA TCATTTTGTGGACGAATAT	-
	KRAS G12A	CATTTTCATTATTTTTATTATAAGGCCTGCTGAAAATGACTGAATATAAACTTGTGG TAGTTGGAGCTGCTGGCGTAGGCAAGAGTGCCTTGACGATACAGCTAATTCAGAAT CATTTTGTGGACGAATAT	-
	KRAS G12D	CATTTTCATTATTTTTATTATAAGGCCTGCTGAAAATGACTGAATATAAACTTGTGG TAGTTGGAGCTGATGGCGTAGGCAAGAGTGCCTTGACGATACAGCTAATTCAGAAT CATTTTGTGGACGAATAT	-
	KRAS G12V	CATTTTCATTATTTTTATTATAAGGCCTGCTGAAAATGACTGAATATAAACTTGTGG TAGTTGGAGCTGTTGGCGTAGGCAAGAGTGCCTTGACGATACAGCTAATTCAGAAT CATTTTGTGGACGAATAT	-
	BRAF WT	CAAACTGATGGGACCCACTCCATCGAGATTTCACTGTAGCTAGACCAAAATCACCTA TTTTTACTGTGAGGTCTTCATGAAGAAATATATCTGAGGTGTAGTAAGTAAAGGAAA ACAGTAGATCTCATTTTCCTATCAGAGCAAGCATTATGAAGAGTTT	-
	BRAF V600E	CAAACTGATGGGACCCACTCCATCGAGATTTCTCTGTAGCTAGACCAAAATCACCTA TTTTTACTGTGAGGTCTTCATGAAGAAATATATCTGAGGTGTAGTAAGTAAAGGAAA ACAGTAGATCTCATTTTCCTATCAGAGCAAGCATTATGAAGAGTTT	-
	NRAS Q61K	CCTGTCCTCATGTATTGGTCTCTCATGGCACTGTACTCTTCTTTTCCAGCTGTATC CAGTATGTCCAACAAACAGGTTTCACCATCTATAACCACTTGTTTTCTGTAAGAATC CTGGGGGTGTGGAGGGTAAGGGGGCAGGGAGGGAGGGAAGTTCAATT	-
	NRAS WT	CCTGTCCTCATGTATTGGTCTCTCATGGCACTGTACTCTTCTTGTCCAGCTGTAT CCAGTATGTCCAACAAACAGGTTTCACCATCTATAACCACTTGTTTTCTGTAAGAAT CCTGGGGGTGTGGAGGGTAAGGGGGCAGGGAGGGAGGGAAGTTCAATT	-
	NRAS Q61R	CCTGTCCTCATGTATTGGTCTCTCATGGCACTGTACTCTTCTCGTCCAGCTGTAT CCAGTATGTCCAACAAACAGGTTTCACCATCTATAACCACTTGTTTTCTGTAAGAAT CCTGGGGGTGTGGAGGGTAAGGGGGCAGGGAGGGAGGGAAGTTCAATT	-

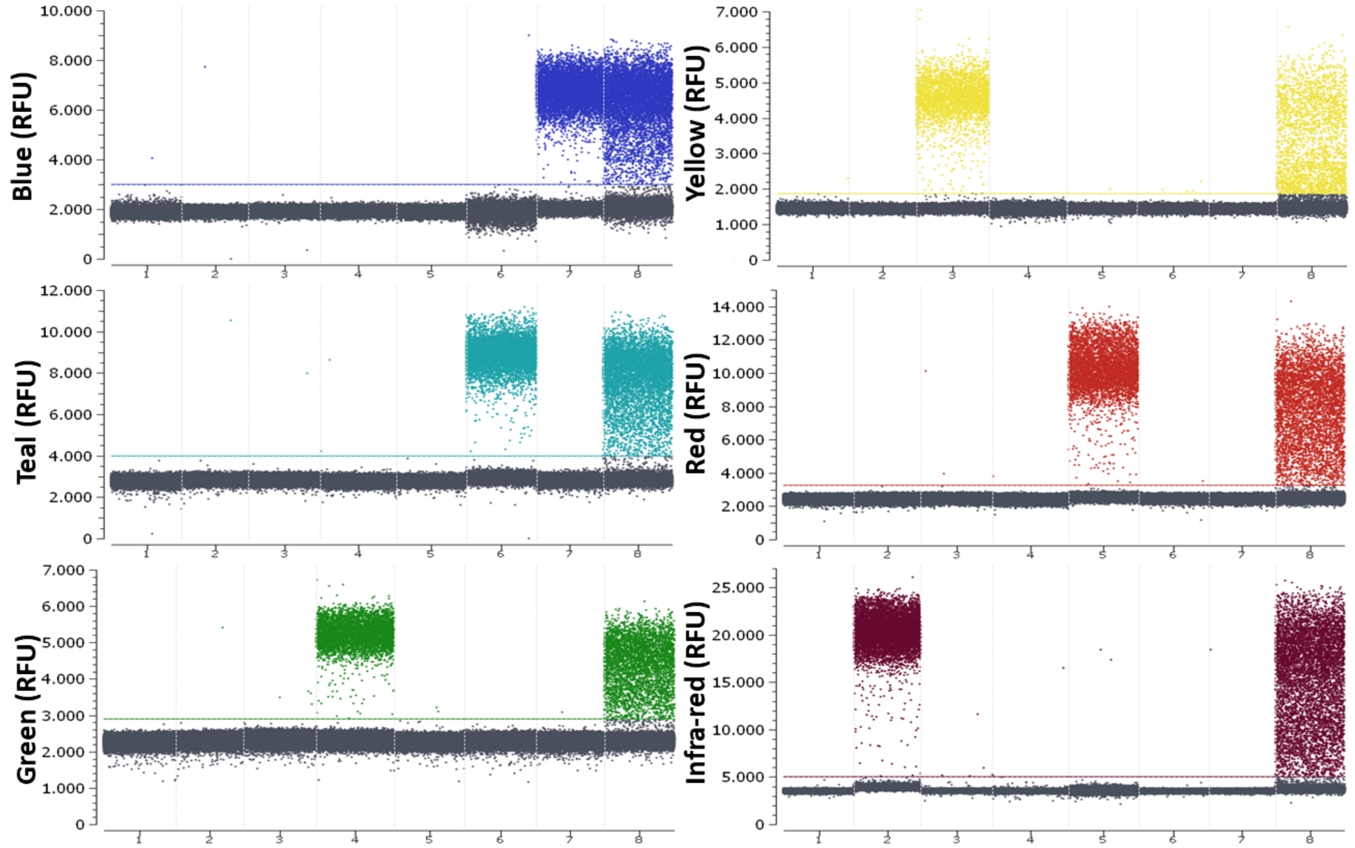

Fluor. Channel \ Chamber	1 NTC	2 *KRAS* WT	3 *KRAS* G12A	4 *KRAS* G12D	5 *KRAS* G12V	6 *BRAF* WT	7 *BRAF* V600E	8 All targets
**Blue**	0	0	0	0	0	0	**10,000 cpr**	**10,000 cpr**
**Teal**	0	0	0	0	0	**10,000 cpr**	0	**10,000 cpr**
**Green**	0	0	0	**10,000 cpr**	0	0	0	**10,000 cpr**
**Yellow**	0	0	**10,000 cpr**	0	0	0	0	**10,000 cpr**
**Red**	0	0	0	0	**10,000 cpr**	0	0	**10,000 cpr**
**Infra-red**	0	**10,000 cpr**	0	0	0	0	0	**10,000 cpr**
**Separability score**	n.a.	11	7	8	7	9	8	From blue to infra-red: 5; 5; 4; 4; 4; 4

Figure S1. Differentiation between positive and negative signal populations in Digital PCR (dPCR) reactions using the 6-plex generic reporter set in combination with a target set for detection of *KRAS* and *BRAF* mutations and their corresponding wildtype (WT) controls. 1 D-plots of all 6 fluorescence channels (labeled "Blue", Teal", "Green", "Yellow", "Red" and "Infra-red") of the naica® Prism6 are shown. Details of the used reporters in each detection channel including sequences, fluorophores and quenchers can be seen in **Table S1** . All detectable droplets formed in eight separate reaction chambers are shown on the x-axis and relative fluorescence intensities (RFU) of each droplet on the y-axis. Reaction chamber details from left to right: (1) no template control (NTC) without target molecule presence; (2-7) 10,000 copies per reaction (cpr) of one single target each; (8) 10,000 cpr of each target combined in one sample. Separability scores between positive and negative droplet populations, as calculated by the Crystal Miner software, are indicated at the bottom of the legend.

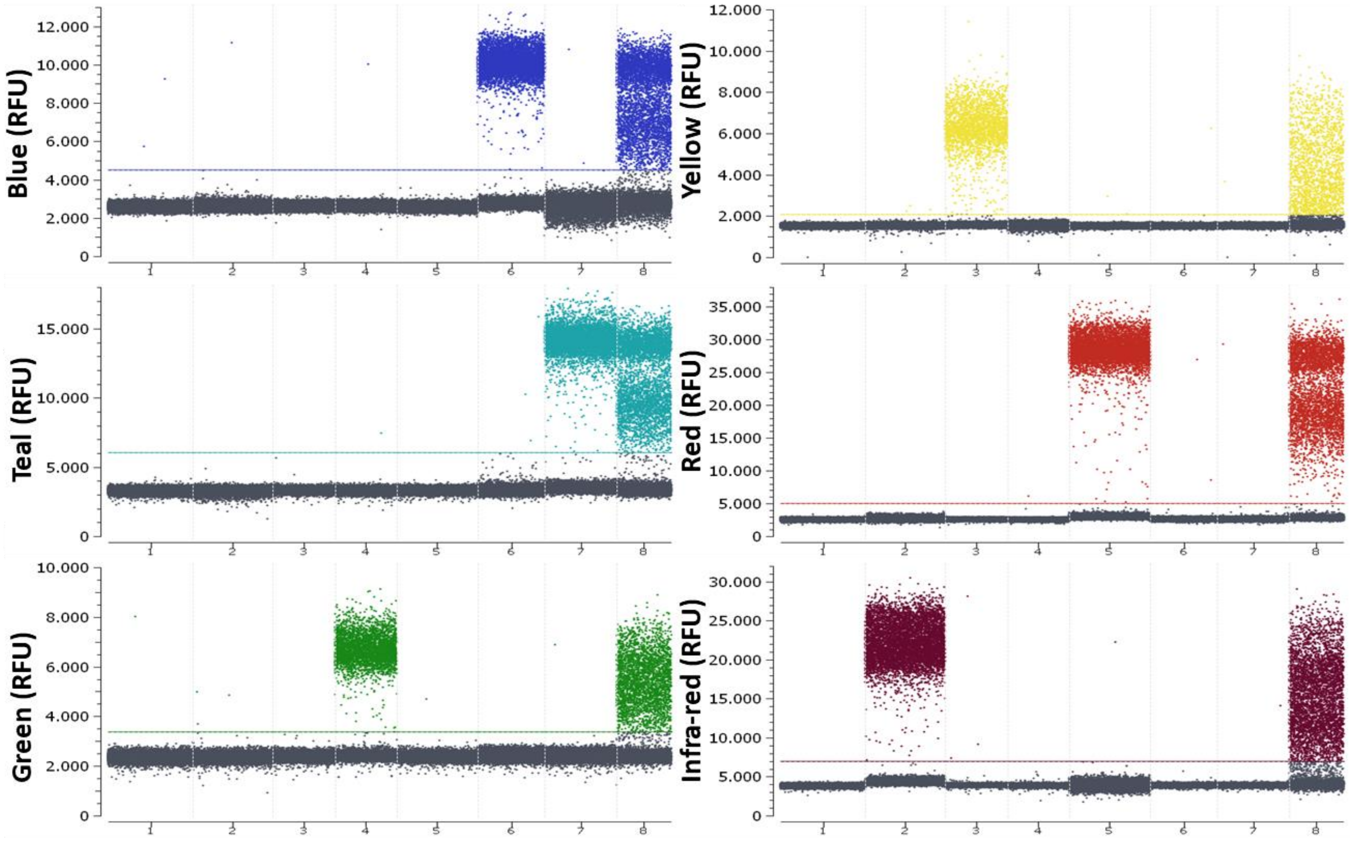

Fluor. Channel \ Chamber	1<br>NTC	2<br>*KRAS*<br>WT	3<br>*KRAS*<br>G12A	4<br>*KRAS*<br>G12D	5<br>N*RAS*<br>Q61K	6<br>*NRAS*<br>WT	7<br>*NRAS*<br>Q61R	8<br>All<br>targets
Blue	0	0	0	0	0	10,000 cpr	0	10,000 cpr
Teal	0	0	0	0	0	0	10,000 cpr	10,000 cpr
Green	0	0	0	10,000 cpr	0	0	0	10,000 cpr
Yellow	0	0	10,000 cpr	0	0	0	0	10,000 cpr
Red	0	0	0	0	10,000 cpr	0	0	10,000 cpr
Infra-red	0	10,000 cpr	0	0	0	0	0	10,000 cpr
Separability score	n.a.	15	6	8	8	12	14	From blue to infra-red: 4; 4; 4; 4; 4; 3

Figure S2. Differentiation between positive and negative signal populations in dPCR reactions using the 6-plex generic reporter set in combination with a target set for detection of *KRAS* and *NRAS* mutations and their corresponding WT controls. 1 D-plots of all 6 fluorescence channels (labeled “Blue”, Teal”, “Green”, “Yellow”, “Red” and “Infra-red”) of the naica® Prism6 are shown. Details of the used reporters in each detection channel including sequences, fluorophores and quenchers can be seen in **Table S1** . All de-tectable droplets formed in eight separate reaction chambers are shown on the x-axis and RFU of each droplet on the y-axis. Reaction chamber details from left to right: (1) NTC; (2-7) 10,000 cpr of one single target each; (8) 10,000 cpr of each target combined in one sample. Separability scores between positive and negative droplet populations, as calculated by the Crystal Miner software, are indicated at the bottom of the legend

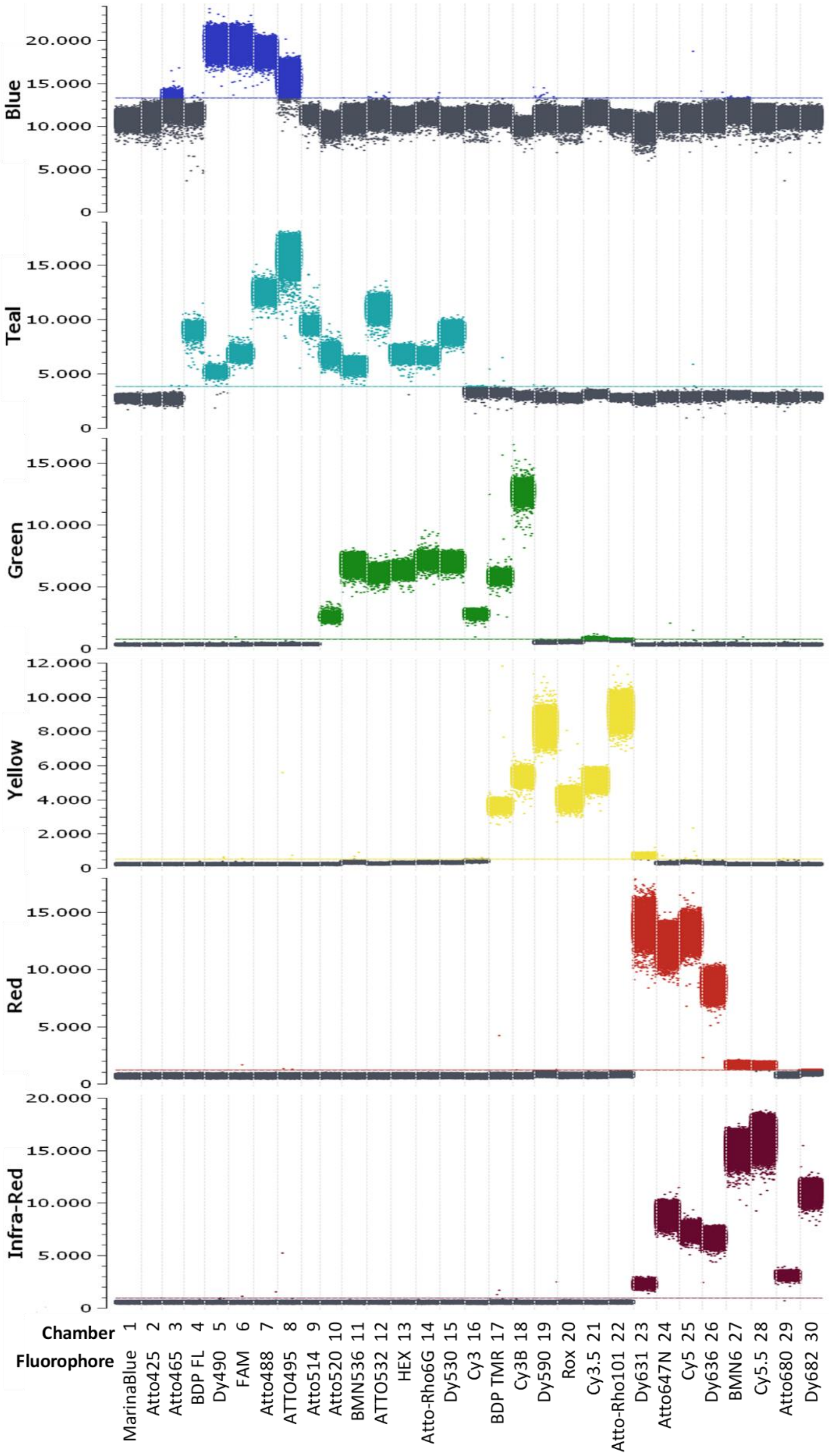

Figure S3. MEA results of 30 reporters, containing each a different fluorophore and a suitable quencher. Exposure times with absorption wavelengths in the naica® Prism6 channels were: blue: 100 ms; teal: 350 ms; green: 100 ms; yellow: 125 ms; red: 500 ms; infra-red: 500 ms. The naica® multiplex PCR MIX 10 X including background fluorophore was used in all reactions. Negative control results (without added mediators) are shown in **Figure S4**.

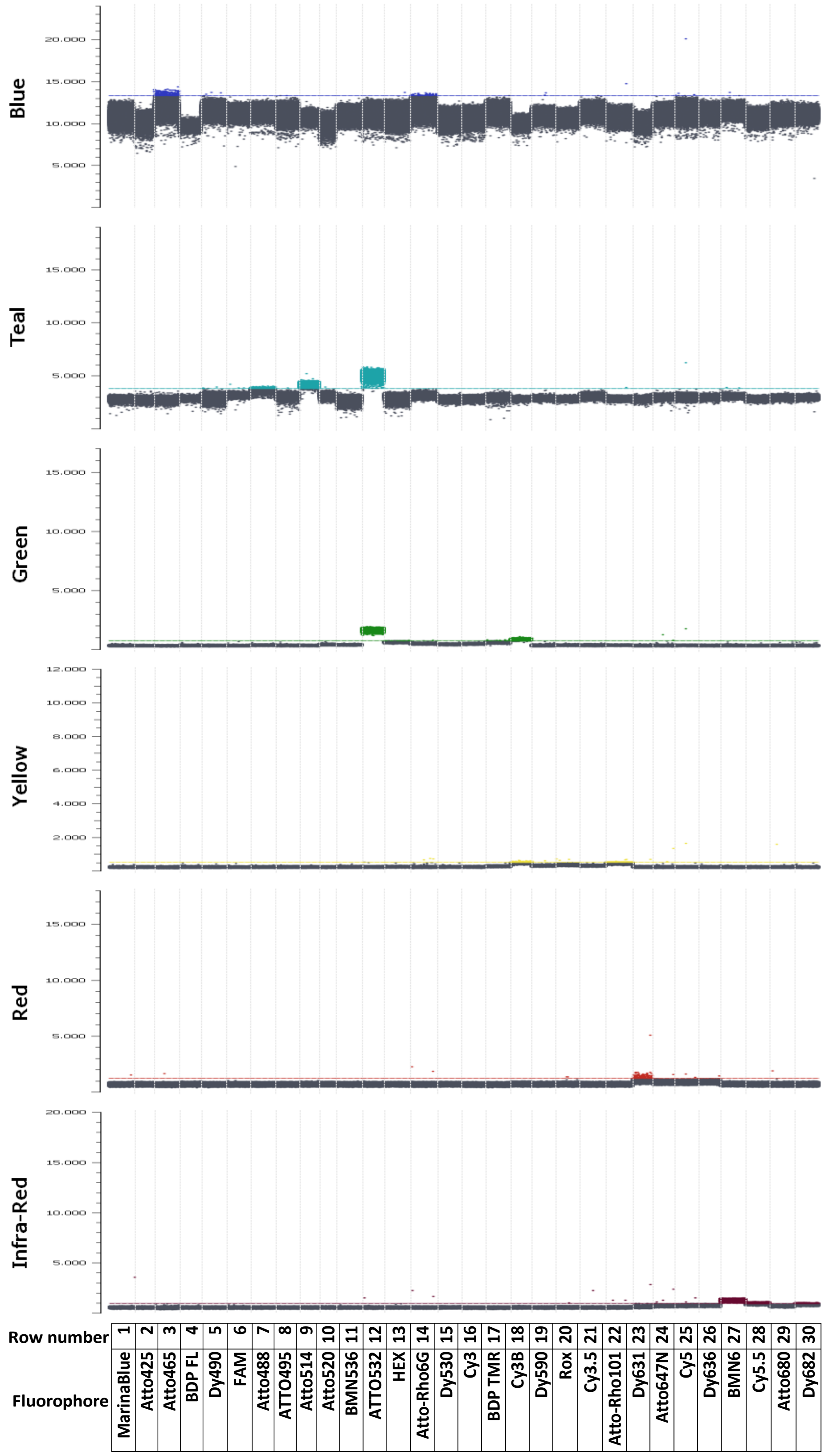

Figure S4. Negative control results (without added mediators) of MEA reactions depicted in **Figure S3.**

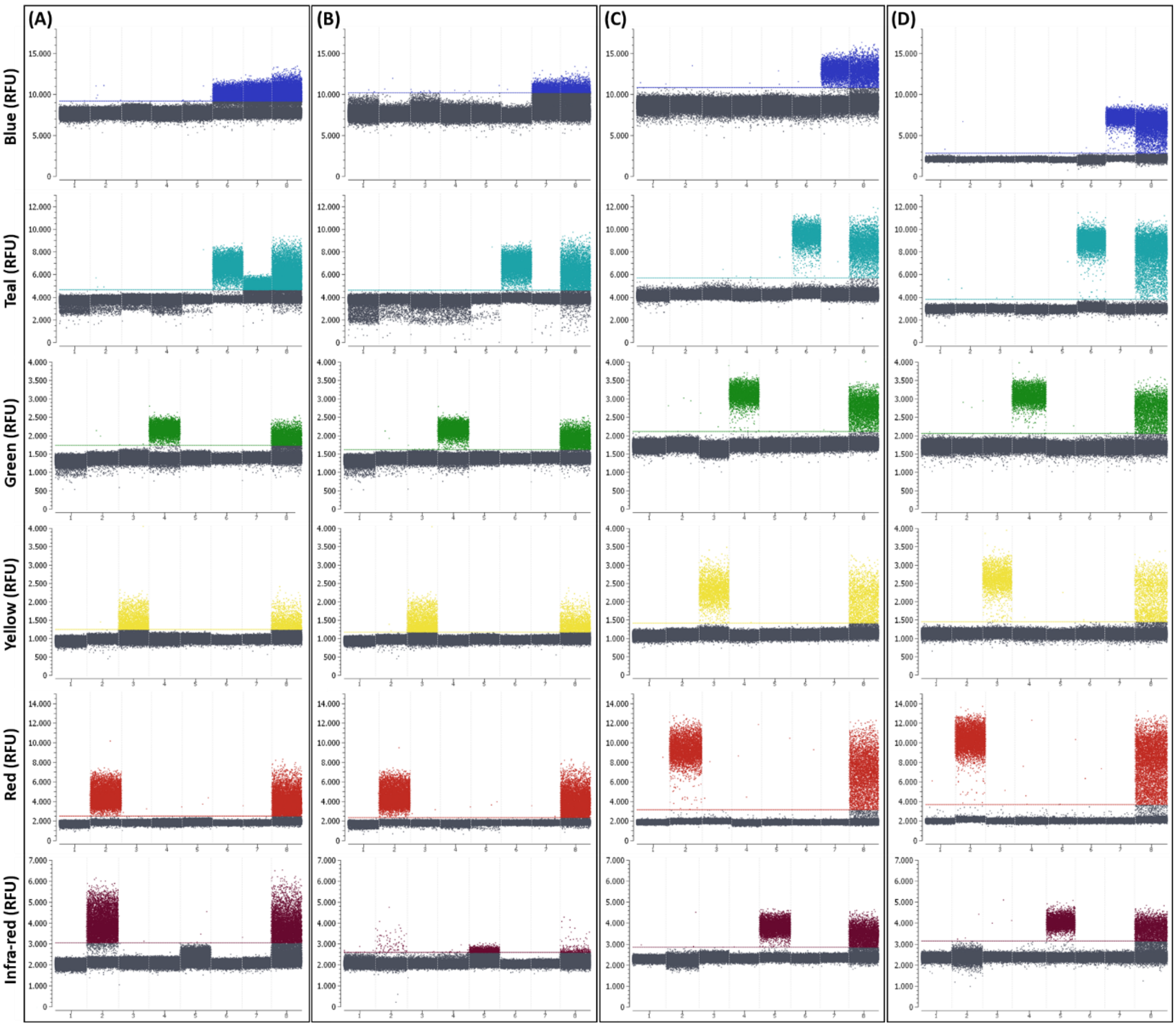

Chamber / Fluor. Channel	1 NTC	2 *KRAS* WT	3 *KRAS* G12A	4 *KRAS* G12D	5 *KRAS* G12V	6 *BRAF* WT	7 *BRAF* V600E	8 All targets
Blue	0	0	0	0	0	0	10,000 cpr	10,000 cpr
Teal	0	0	0	0	0	10,000 cpr	0	10,000 cpr
Green	0	0	0	10,000 cpr	0	0	0	10,000 cpr
Yellow	0	0	10,000 cpr	0	0	0	0	10,000 cpr
Red	0	10,000 cpr	0	0	0	0	0	10,000 cpr
Infra-red	0	0	0	0	10,000 cpr	0	0	10,000 cpr
Separability score (A)	n.a.	3	2	4	4	5	3	From blue to infra-red: 3; 2; 3; 2; 3; 2
Separability score (B)	n.a.	2	2	4	6	4	3	From blue to infra-red: 2; 3; 3; 2; 3; 2
Separability score (C)	n.a.	8	4	7	5	7	4	From blue to infra-red: 3; 5; 4; 3; 4; 3
Separability score (D)	n.a.	9	6	6	6	9	10	From blue to infra-red: 5; 5; 4; 4; 4; 3

Figure S5. Improving discriminability between positive and negative signal populations in dPCR reactions with increasing assay optimization level. During all steps of generic reporter set optimization, the target set for detection of *KRAS* and *BRAF* mutations and their corresponding WT controls was used. 1 D-plots of all 6 fluorescence channels of the naica® Prism6 are shown. Reaction chamber details from left to right: (1) NTC; (2 - 7) 10,000 cpr of one single target each; (8) 10,000 cpr of each target combined in one sample. Separability scores between positive and negative droplet populations, as calculated by the Crystal Miner software, are indicated at the bottom of the legend. Fluorophores for this generic reporter set were selected according to the MEA data shown in **Figure S3**. Details of the used reporters in each detection channel including sequences, fluorophores and quenchers can be seen in **Table S2**. (A) Initial non-optimized test without color compensation. (B) Initial non-optimized test after setting a suitable color compensation, with which each target is detected in only one fluorescence channel. This

color compensation matrix was henceforth used in every subsequent test to optimize the generic reporter set. (C) Improved separability after increasing the PCR cycle number from 45 to 60. (D) Further improved separability after reduction of background fluorescence in the "Blue" and "Teal" channels by changing the naica® mastermix version from "naica® multiplex PCR MIX" to "naica® PCR MIX".

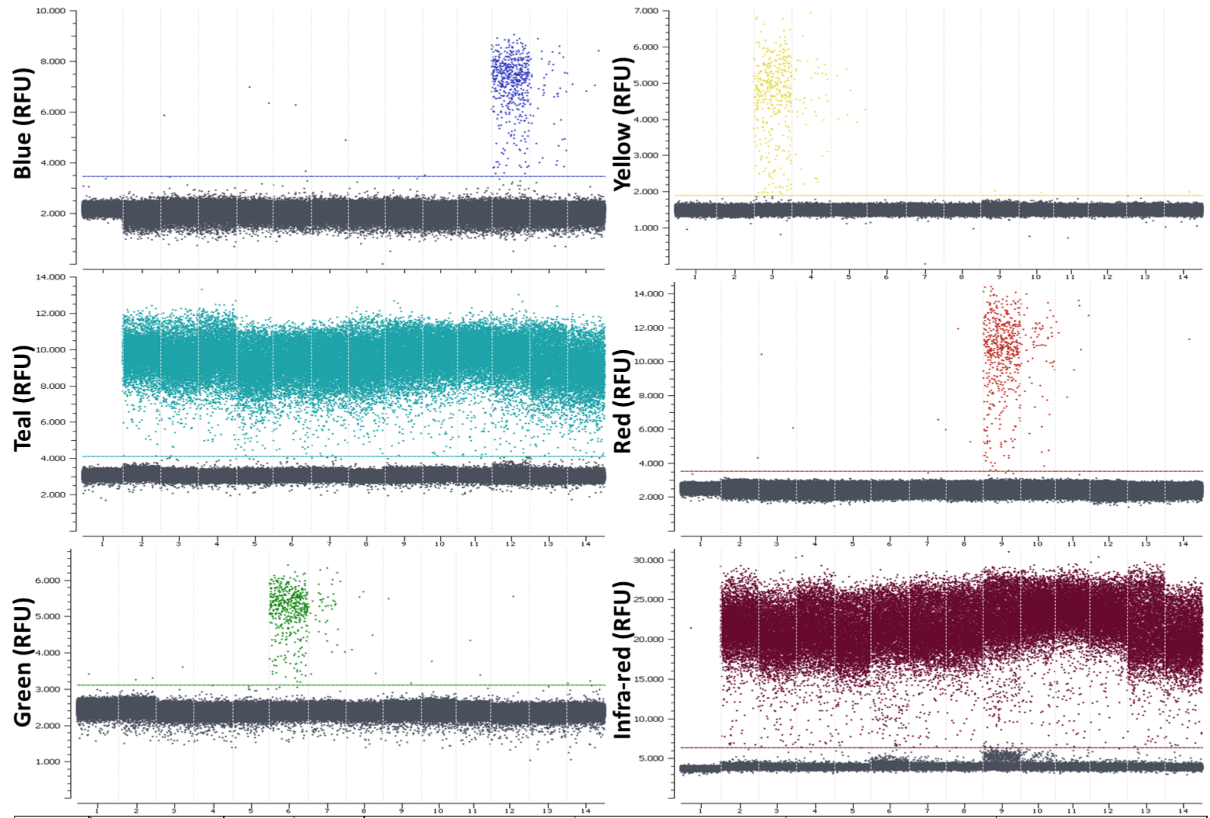

Fluor. Channel	Chamber / Parameter	1 NTC	2 Only WTs	3-5 *KRAS* G12A dilutions + WTs			6-8 *KRAS* G12D dilutions + WTs			9-11 *KRAS* G12V dilutions + WTs			12-14 *BRAF* V600E dilutions + WTs		
**Blue**	**Expected (cp/µl)**	**0**	**0**	**0**	**0**	**0**	**0**	**0**	**0**	**0**	**0**	**0**	**40**	**4**	**0.4**
	**Measured (cp/µl)**	**0±0**	**0±0**	**0.1±0**	**0±0**	**0.1±0**	**0±0**	**0±0**	**0.1±0.1**	**0±0**	**0±0**	**0±0**	**35.8±3.9**	**4.1±0.4**	**0.4±0.2**
	Positive droplets	0±1	0±0	2±1	0±1	1±1	1±0	0±0	1±1	0±0	1±1	1±1	493±47	52±2	6±3
**Teal**	**Expected (cp/µl)**	**0**	**400**	**400**	**400**	**400**	**400**	**400**	**400**	**400**	**400**	**400**	**400**	**400**	**400**
	**Measured (cp/µl)**	**0±0**	**395.3±7.9**	**393.6±3.5**	**384.9±14.8**	**374±6.5**	**380.5±5.7**	**390.7±13.6**	**382.8±4.3**	**384.1±15.8**	**385.1±20.6**	**404.3±13.9**	**390.7±22.6**	**380±34.3**	**381.2±16.6**
	Positive droplets	0±0	4636±464	4508±348	4169±967	4440±405	4518±332	4338±372	4756±229	4551±315	4405±104	4461±26	4837±170	4301±338	4318±394
**Green**	**Expected (cp/µl)**	**0**	**0**	**0**	**0**	**0**	**40**	**4**	**0.4**	**0**	**0**	**0**	**0**	**0**	**0**
	**Measured (cp/µl)**	**0±0**	**0±0**	**0±0**	**0±0**	**0±0**	**33.4±2.8**	**2.8±0.3**	**0.2±0**	**0±0**	**0.1±0**	**0±0**	**0±0**	**0±0**	**0±0**
	Positive droplets	0±0	1±1	0±0	0±0	0±0	444±72	35±6	4±1	1±1	1±0	1±1	0±0	0±1	0±1
**Yellow**	**Expected (cp/µl)**	**0**	**0**	**40**	**4**	**0.4**	**0**	**0**	**0**	**0**	**0**	**0**	**0**	**0**	**0**
	**Measured (cp/µl)**	**0±0**	**0±0**	**24.5±5**	**2.3±0.6**	**0.6±0.4**	**0±0**	**0±0**	**0±0**	**0±0**	**0±0**	**0±0**	**0±0**	**0±0**	**0±0**
	Positive droplets	0±1	0±1	316±77	29±12	8±4	0±0	0±0	0±0	0±0	0±0	0±0	0±0	1±1	0±0
**Red**	**Expected (cp/µl)**	**0**	**0**	**0**	**0**	**0**	**0**	**0**	**0**	**40**	**4**	**0.4**	**0**	**0**	**0**
	**Measured (cp/µl)**	**0±0**	**0±0**	**0±0**	**0±0.1**	**0±0**	**0±0.1**	**0±0**	**0±0**	**37.4±3.4**	**3.4±1.4**	**0.5±0.1**	**0±0**	**0±0**	**0±0**
	Positive droplets	0±0	0±0	1±1	1±1	0±0	1±1	0±1	1±1	494±75	44±16	7±1	0±0	0±0	0±0
**Infra-red**	**Expected (cp/µl)**	**0**	**400**	**400**	**400**	**400**	**400**	**400**	**400**	**400**	**400**	**400**	**400**	**400**	**400**
	**Measured (cp/µl)**	**0.1±0**	**409.8±15.7**	**422.5±16.9**	**407±28.7**	**395.3±26.4**	**404.9±34.5**	**422±33.3**	**415.6±28.1**	**410.1±22.3**	**422.3±9.3**	**425.7±29.8**	**417.4±19.3**	**396.9±40**	**401.9±30.8**
	Positive droplets	1±0	4776±344	4788±218	4334±731	4659±465	4759±300	4627±221	5118±496	4814±178	4783±214	4668±297	5132±300	4459±161	4507±130
**Separability score**		n.a.	9 (Teal); 9 (IR)	6	5	5	7	5	3	6	6	5	7	5	7

Figure S6. Quantitative dPCR results of the 6-plex generic reporter set in combination with a target panel for detection of *KRAS* and *BRAF* mutations and their corresponding WT controls. 1 D-plots of all 6 fluorescence channels detecting either WTs (in "Teal" and "Infra-red") or mutations (in "Blue", "Green", "Yellow" and "Red"). A background of 10,000 cpr of both WT targets is present in all reaction chambers aside from the NTC (reaction chamber 1). Single mutation targets were added in three different concentrations (1,000 cpr, 100 cpr and 10 cpr). Quantitative data of three biological replicates are depicted in the figure legend. Expected concentrations and mean values and standard deviations of measured concentrations in copies per microliter (cp / µl) as well as mean values and standard deviations of the number of positive droplets are shown for each reaction chamber in each detection channel. Cells are color-coded, whenever a positive result was expected for the specific chamber and channel. Separability scores between positive and negative droplet populations in the depicted reactions, as calculated by the Crystal Miner software, are indicated at the bottom.

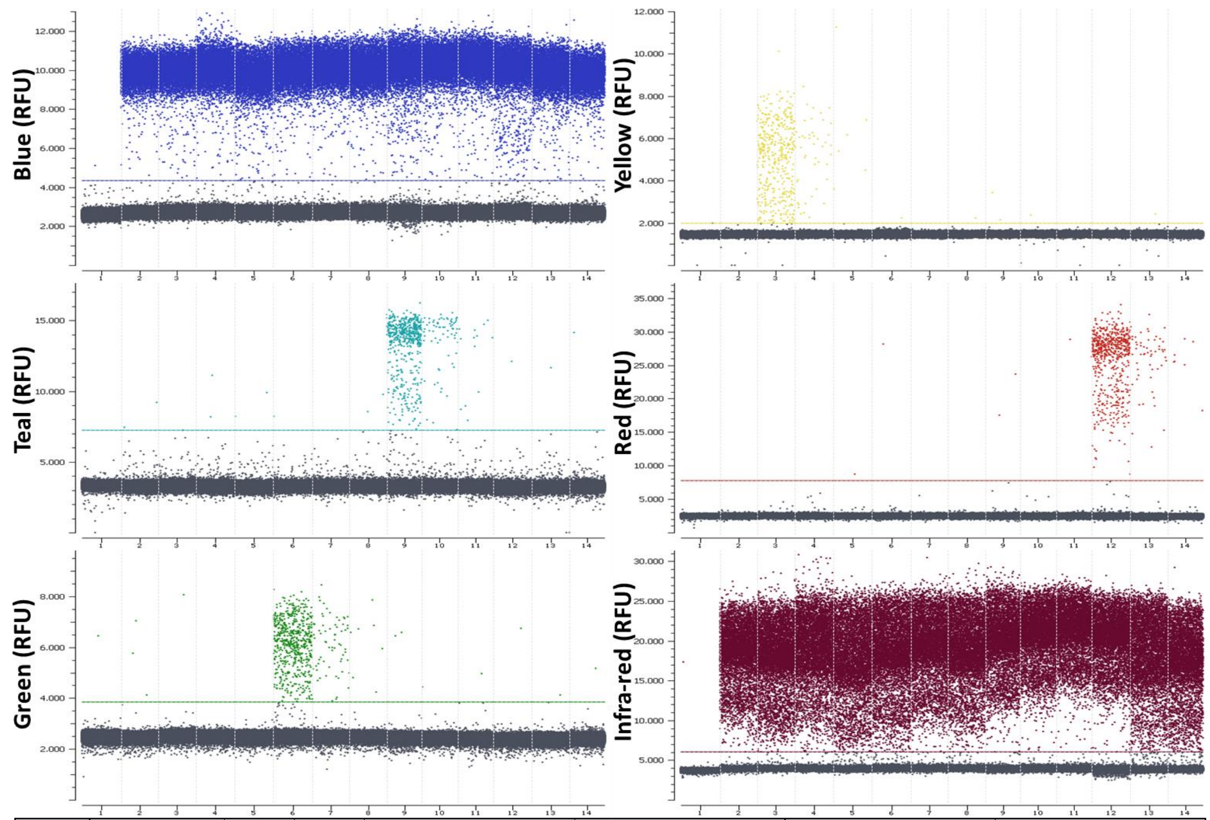

Fluor. Channel	Chamber / Parameter	1 NTC	2 Only WTs	3-5 *KRAS* G12A dilutions + WTs			6-8 *KRAS* G12D dilutions + WTs			9-11 *NRAS* Q61R dilutions + WTs			12-14 *NRAS* Q61K dilutions + WTs		
Blue	**Expected (cp/µl)**	**0**	**400**	**400**	**400**	**400**	**400**	**400**	**400**	**400**	**400**	**400**	**400**	**400**	**400**
	**Measured (cp/µl)**	**0.1±0**	**429.5±4.3**	**437.8±12.3**	**433±17.9**	**418.9±8.6**	**433.1±11.2**	**433±7.5**	**426.9±6.1**	**427±9.8**	**430.6±19.9**	**442.6±16.8**	**443.1±7.2**	**428.7±21.2**	**430.5±18.2**
	Positive droplets	1±0	5098±254	5182±380	5391±151	4932±443	5102±430	4991±308	4917±303	5051±149	5072±313	4911±390	5165±296	4584±797	4344±742
Teal	**Expected (cp/µl)**	**0**	**0**	**0**	**0**	**0**	**0**	**0**	**0**	**40**	**4**	**0.4**	**0**	**0**	**0**
	**Measured (cp/µl)**	**0±0**	**0.1±0**	**0±0**	**0±0**	**0.1±0**	**0.1±0**	**0±0.1**	**0.1±0**	**37.1±6.1**	**3.7±1.2**	**0.6±0.2**	**0.1±0**	**0±0**	**0±0**
	Positive droplets	0±0	2±0	0±0	0±1	2±0	1±0	1±1	1±1	492±55	50±17	8±3	1±0	0±0	0±0
Green	**Expected (cp/µl)**	**0**	**0**	**0**	**0**	**0**	**40**	**4**	**0.4**	**0**	**0**	**0**	**0**	**0**	**0**
	**Measured (cp/µl)**	**0±0**	**0±0.1**	**0±0**	**0±0**	**0.1±0.1**	**35.1±5.1**	**3.9±0.8**	**0.5±0.1**	**0.1±0**	**0±0**	**0.1±0**	**0±0**	**0.1±0**	**0.1±0**
	Positive droplets	1±0	1±1	1±1	1±1	1±1	469±93	51±13	7±2	2±0	1±0	1±1	1±0	1±0	1±0
Yellow	**Expected (cp/µl)**	**0**	**0**	**40**	**4**	**0.4**	**0**	**0**	**0**	**0**	**0**	**0**	**0**	**0**	**0**
	**Measured (cp/µl)**	**0±0**	**0±0**	**28.1±6.6**	**2.6±0.9**	**0.4±0**	**0±0**	**0±0**	**0±0**	**0±0**	**0±0**	**0±0**	**0±0**	**0±0**	**0±0**
	Positive droplets	0±1	0±0	380±105	37±13	6±0	0±0	0±0	1±1	1±1	0±0	0±0	0±0	0±0	0±0
Red	**Expected (cp/µl)**	**0**	**0**	**0**	**0**	**0**	**0**	**0**	**0**	**0**	**0**	**0**	**40**	**4**	**0.4**
	**Measured (cp/µl)**	**0±0**	**0±0**	**0±0**	**0±0**	**0±0**	**0±0**	**0±0**	**0±0**	**0.1±0**	**0±0**	**0.1±0**	**39.2±4.7**	**3.6±0.3**	**0.4±0.1**
	Positive droplets	0±0	0±1	0±0	0±0	0±0	1±0	0±0	0±0	1±0	0±0	1±0	520±95	45±12	5±2
Infra-red	**Expected (cp/µl)**	**0**	**400**	**400**	**400**	**400**	**400**	**400**	**400**	**400**	**400**	**400**	**400**	**400**	**400**
	**Measured (cp/µl)**	**0±0**	**409.8±16.5**	**421.6±8.4**	**415.4±8.6**	**408.6±13.9**	**414.6±10.1**	**412.5±22.5**	**413.6±1.1**	**410.8±14.9**	**420±16.1**	**431.4±22.1**	**427.4±11.6**	**399.7±10.6**	**410.4±14.6**
	Positive droplets	1±0	4892±267	5011±290	5202±159	4816±294	4910±388	4780±336	4784±310	4883±189	4962±271	4802±404	5004±295	4318±785	4166±709
**Separability score**		n.a.	13 (Blue); 6 (IR)	12	11	10	5	4	5	7	9	5	7	5	5

Figure S7. Quantitative PCR results of the 6-plex generic reporter set in combination with a target panel for detection of *KRAS* and *NRAS* mutations and their corresponding WT controls. 1 D-plots of all 6 fluorescence channels detecting either WTs (in "Blue" and "Infra-red") or mutations (in "Teal", "Green", "Yellow" and "Red"). A background of 10,000 cpr of both WT targets is present in all reaction chambers aside from the NTC (reaction chamber 1). Single mutation targets were added in three different concentrations (1,000 cpr, 100 cpr and 10 cpr). Quantitative data of three biological replicates are depicted in the figure legend. Expected concentrations and mean values and standard deviations of measured concentrations in cp / µl as well as mean values and standard deviations of the number of positive droplets are shown for each reaction chamber in each detection channel. Cells are color-coded, whenever a positive result was expected for the specific chamber and channel. Separability scores between positive and negative droplet populations in the depicted reactions, as calculated by the Crystal Miner software, are indicated at the bottom.
